# Comprehensive multivariate correlations between climatic effect, metabolite-profile, antioxidant capacity and antibacterial activity of Brazilian red propolis metabolites during seasonal study

**DOI:** 10.1038/s41598-019-54591-3

**Published:** 2019-12-04

**Authors:** Ticiano Gomes do Nascimento, Rodolfo Elleson dos Santos Arruda, Erika Tayse da Cruz Almeida, José Marcos dos Santos Oliveira, Irinaldo Diniz Basílio-Júnior, Isabel Cristina Celerino de Moraes Porto, Adilson Rodrigues Sabino, Josealdo Tonholo, Alexander Gray, RuAngelie Edrada Ebel, Carol Clements, Tong Zhang, David George Watson

**Affiliations:** 1 Laboratory of Pharmaceutical and Food Analysis, Postgraduate Program in Pharmaceutical Sciences, Institute of Pharmaceutical Sciences and Postgraduate program of Nutrition, College of Nutrition, Federal University of Alagoas, AC Simões Campus, University City, Maceió, Alagoas 57072 -970 Brazil; 2 University of Strathclyde, Department of Pharmaceutical Sciences, Strathclyde Institute of Pharmacy and Biomedical Science, 27 Taylor Street, Glasgow, G4 0NR UK

**Keywords:** Metabolomics, Drug safety, Natural products, Antiparasitic agents

## Abstract

The standardization of apiceutical products like as propolis extracts has been widely debated worldwide and variations in the propolis chemical composition are still very relevant topics for use-standardized of different propolis-type as medication by much of the world’s population. The present manuscript discuss important issues related to the climate effect and variations in propolis metabolite-profiling changes, antioxidant capacity and variations of the antibacterial activity of the Brazilian red propolis metabolites using comprehensive multivariate correlations. It was observed the increasing of guttiferones concentrations during the intense drought period and drastic decreasing in rainy period. The climate variation induced the high concentration of flavonoids in rainy period with pronounced dropped in some rainy months. The Pearson´s analysis demonstrated correlation between IC_50_ from DPPH and guttiferones and flavonoids concentrations. The PCA-X and Hotelling T2 test showed outliers during the months with lowest concentrations of formononetin and isoliquiritigenin was observed in antibacterial tests. The PLS-DA, OPLS-DA and VIP analysis demonstrate guttiferone E, guttiferone B, liquiritigenin, naringenin are considered important substances responsible by anti-staphylococcal activity in red propolis composition during the rainy season and drought period, but a synergistic effect with other flavonoids and isoflavonoids are not ruled out.

## Introduction

The Brazilian red propolis (BRP) is common raw material produced by the bees and found in mangrove areas of the State of Alagoas, Brazil and the main botanical source is the “rabo de bugio” (*Dalbergia ecastophyllum (L)* Taud). According to the Brazilian propolis classification, the BRP is classified into 13 Group and can be distinguished from the others propolis, due to the presence phenolic compounds such as: isoflavones, chalcones, isoflavones, pterocarpans, terpenes, polyprenylated benzophenones (guttiferones), condensed tannins, and others^1–5^.

Seasonality studies are usually conducted as part of a program of standardization and qualification of raw material and apiceutical products. Researchers worldwide have discussed the challenge of standardization of apiculture products and based on propolis raw material^6,7^, some factors are known like as geographical area^3,6–8^, vegetation type and botanical origin^1–3,8,9^,0, climate changes with heat wave and prolonged droughts^10–12^, weather^10–12^, type of bee and queen-bee genetics^10–14^, hives management^12^, harvesting time^12^. However, the problem of standardization and variability remain the same in decades of study^6–8^.

The advances in analytical techniques to evaluate the authenticity, chemical profiling and isolation of constituents in propolis are being modernized and GC-MS and LC-MS technique has presented great applicability for this purpose^5,15–17^. The chromatographic methods for instrumental analysis of propolis, honey and botanical drugs should be sensitive, specific, and reproducible and, be able to perform a global analysis discriminating it from possible contaminants in these materials. For detection and determination of these constituents, modern chromatographic methods combined with mass spectrometry, such as LC-Orbitrap-FTMS, has proven to be a suitable alternative for analysis of propolis because it allows to performing a comprehensive analysis of apiceutical products^5,15,16^.

In particular, the development of seasonal studies when properly delineated has great advantages and can contribute with the establishment of the chemical composition of the commercial propolis^18,19^. It establishes the best times of the year for the harvesting of propolis with excellent quality^20^; it avoids the falsification or the purchase of propolis raw material in low quality^21^ and it establishes indicators minimum quality from commercial point view^21–23^. In research, the seasonality can assists and facilitates phytochemical research to identify interest chemical compounds during the discovery of medicinal substances when coupled with universal detector during fingerprint studies^23^.

At present, a few studies developed to verify and understand the variation of phenolic compounds and biological activities influenced in most case by the climate variations^24–29^, and there is no studies to establish some meteorological-chemical-biological correlations of propolis extracts were purpose as quality control tool for propolis raw materials. This seasonality study will demonstrate how climatic variations can affect the concentrations of some phenolic compounds of red propolis and their interrelations with antioxidant and biological activities. The aim of the present work was to stablish comprehensive multivariate correlations between the climate effect and variation of propolis metabolite-profiling changes, antioxidant capacity, composition and variations of the antibacterial activity of the Brazilian red propolis metabolites in northeast area (Alagoas State) from Brazil.

## Results and Discussion

### Fingerprint of red propolis extracts using LC-ESI-Orbitrap-FTMS

The red propolis extracts (propolis A, B and C) presented a similar chromatographic profile in the retention time range for flavonoids between 5 and 22 minutes and variations in the range between 22 and 50 minutes corresponding to the identification of terpenes, propolones and guttiferones (see Fig. S1).The extracts of Brazilian red propolis already have a known chemical composition. Several researchers have identified several flavonoids, isoflavonoids, pterocarpans, terpenes, guttiferones in Brazilian red propolis. A large majority of substances present in Brazilian red propolis are not commercially available and were isolated in the laboratory like as: retusapurpurin A and B, xanthochymol, vestitol and neo-vestiol, and new chalcones^5,30–33^.

Our research group has been performing chromatographic profile and fingerprint studies of Brazilian red propolis. Several important flavonoids have been identified in red propolis such as liquiritigenin, pinobanksin, galangin, isoliquiritigenin, formononetin, daidzein, medicarpin, guttiferone E, guttiferone B among others^5,34^ (see Table S1). Based on these fingerprint studies, some available commercially flavonoids and two laboratory isolated guttiferones were chosen to monitor the concentration of phenolic compounds present in the red propolis, thus assessing variabilities over a 1-year cycle, assessing their relationship with the climate and also variability of biological activities.

### Determination of flavonoids and other phenolic using LC-DAD-UV and LC-Orbitrap-FTMS

Flavonoid concentration obtained using LC-UV-DAD and LC-ESI-Orbitrap-FTMS methods have demonstrated a seasonal profile similar to classical UV-vis and total phenol methods using Folin-Ciocalteu. Despite the same seasonal profile obtained by the two chromatographic methods, an overestimation of the flavonoid concentration was observed when LC-UV-DAD determination was performed (see Figs. S2 and S3). This was observed due to some co-elution problems during the analytical runs. The use of quercetin and chrysin as internal standard were important to stablish the retention time of the flavonoids in quantitation analysis. The apiaries presented its own variability in the seasonal profile. However, in all apiaries, a common variation (between May and September) it was observed, with an increase in the concentration of the major flavonoids (Liquiritigenin, isoliquiritigenin and formononetin), as well as in the concentration of the other flavonoids of low concentrations present in red propolis (Biochanin A, genistein, daidzein, luteolin, naringenin, galangin, pinobanksin, pinocembrin). This increase in the concentration of flavonoids coincides with the period of intense rainfall in the Alagoas State of the Brazilian northeast and had a strong correlation with the meteorological data of the National Institute of Meteorology of the Brazilian government^35^ (www.inmet.gov.br).

**Figure 1:**
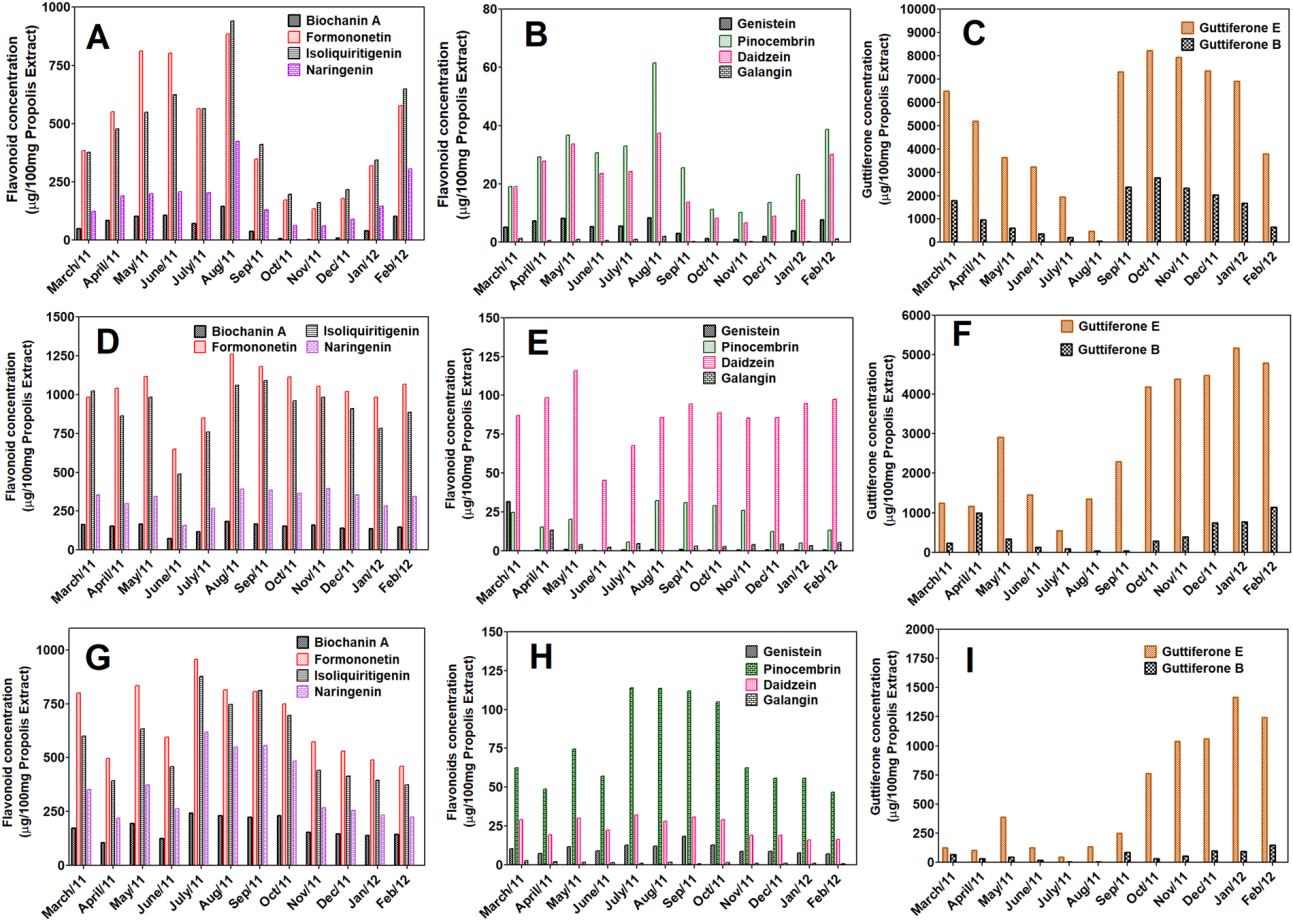
Determination of the isoflavonoids, flavonoids and guttiferones of the Brazilian red propolis extracts using LC-Orbitrap-FTMS. Propolis **A** (**A–C**), Propolis **B** (**D–F**) and Propolis **C** (**G–I**). The concentrations were expressed as amount in microgram of flavonoids or guttiferones present in 100 mg of red propolis extract.

Other important discussion were the variations on the concentration observed in the quantification of guttiferones E and B during the seasonal studies using the LC-ESI-Orbitrap-FTMS method (Fig. 1). During the studied annual cycle (March/2011 to February/2012), the guttiferones E and B presented a low concentration during periods of intense rains and an increase in the concentration of these substances in the period between October/2011 and March/2012, in summer with intense sunlight. Although these substances belong to the class of phenolic compounds and should therefore follow the same pattern of variation on the concentration similar to flavonoids during the period of intense rains, this fact did not occur, except for Primavera apiary in punctual months (June and July).

### Analytical methods correlation

The Table S3 shows rainfall data in time series between 2009 and 2019. We can observe that during the study period (cycle 2011–2012), a period of intense rains occurred between April and August. Occasional rains also occurred in January, March and September (see Table S3). For the other years of this time series, there is variation in the beginning of the rains and also in its intensity. The time series 2009 to 2019 shows that there is a greater variability of rainfall between January and March with rainfall ranging from 8 mm (March/2011) to 220 mm (February/2018) (see Table S3). We can also observe that in the time series, there are irregularities of rains even in the period considered rainy; since we must also associate, it with the phenomena El Niño and La Niña that occurs in alternate periods (see Fig. S5). The periods from 2007 to 2009 and 2010 to 2012 we saw the effect of La Niña, while from 2014 and greater intensification between 2015 and 2016 occurred the effect of El Niño^36^. As for solar radiation (see Fig. S4 and Table S4), we also presented a time series from 2011 to 2012 and observed some solar variations, we noticed changes in the rainy period with solar radiation ranging between 500–750 kJ/m^2^ (2011) in the period from April to September and 1712 kJ/m^2^ during December (2011) and 1155 kJ/m^2^ during February (2012). We can also observe that the planet has undergone two solar storms in the year of 2012 and announcing by the site BBC News^37^. So, we can say that the variations of rain intensities and solar radiation vary each year. These variations may be reflecting on the composition and concentration of the phenolic compounds present in the plants used by the bees to produce red propolis, consequently promoting variations in the composition and phenolic content of the raw material red propolis.

**Figure 2:**
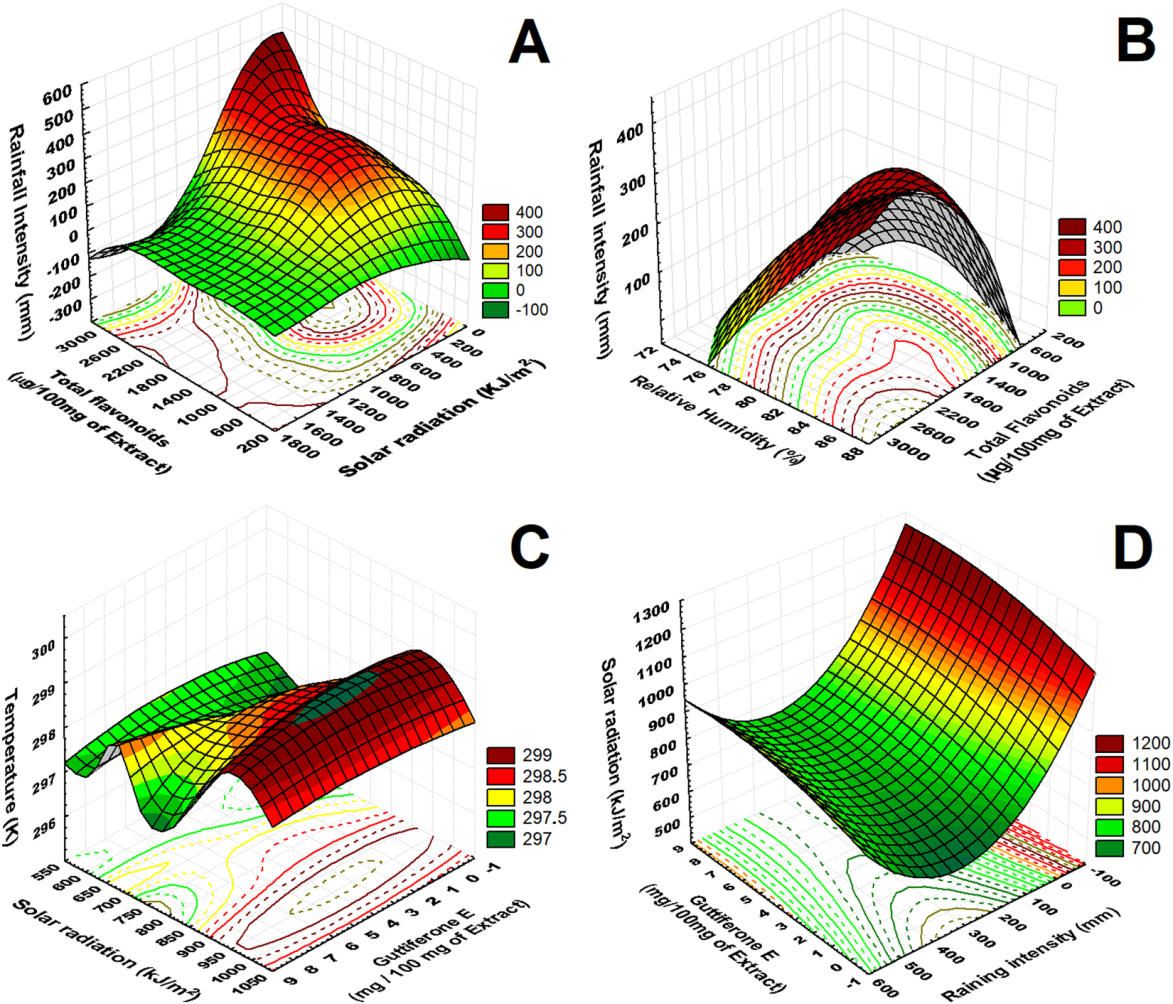
Correlation on the 3D surface graphs (XYZ) between phenolic compounds concentration and meteorological data. (**A**) flavonoids concentration in µg/100 mg of propolis extract (X) versus rainfall intensity in mm (Y) and solar radiation (Z). (**B**) flavonoids concentration µg/100 mg of propolis extract (X) versus rainfall intensity (Y) and relative humidity (Z). (**C**) Guttiferone E concentration µg/100 mg of propolis extract (X) versus temperature in Kelvin (Y) and solar radiation (Z). (**D**) Guttiferones E concentration µg/100 mg of propolis extract (X) versus solar radiation in kJ.m^2^ (Y) and rainfall intensity in mm (Z).

The meteorological data provides the basis for the direct relation of the concentration of flavonoids with the climate changing. When the proportion of rainfall and humidity is increased, it was noticed that the amount of flavonoids is directly related to rainfall and relative humidity intensities. The optimal concentration of flavonoids is related to the shorter solar radiation (200–1000 kJ/m^2^). So, the better climate parameters to establish a high concentration of flavonoids were the high intensity of rainfall relative humidity and occurred in the months between June, July, August, and September where rainfall intensity increases. There is an increase in the concentration of guttiferones E, when the incidence of sunlight and temperature increase, and this increase occurs between the months of November and March (drought period), so in months where there were average temperatures below 30 °C and incidence of sunlight (750–1360 kJ/m^2^) with optimal condition between (900–1000 kJ/m^2^) (Fig. 2). The concentration of this component was detected as previously observed in the apiaries studied. Decreased concentration of guttiferone E was observed with increasing rainfall intensity during and decreasing in the intensity solar radiation (Fig. 2).

The comparison between the analytical methods was performed through the Pearson´s correlation (ρ) matrix analysis, as well as the normalization of the concentrations of the different methods tested. The correlation between the analytical methods used for quantification of flavonoids was performed using matrix scatter statistics and Pearson’s correlation (ρ). The methods of chemical and spectrophotometric reaction (total phenolic method and UV-vis) (correlation 0.62 > ρ > 0.78), as well as chromatographic methods (0.63 > ρ > 0.98) showed a good correlation with values above 60% correlation. We also observed a good correlation between total phenolic methods and LC-DAD (0.60 > ρ > 0.61) and between UV-vis and LC-DAD (0.65 > ρ > 0.75). Weak correlation between LCMS with total phenols and UV-vis, only a single sample correlation >0.60 (see Fig. S6 and Table S6).

In our experiments, the quantitative analyses were started by the general methods like as Folin-Ciocalteu and UV-vis, which aroused interest in proceeding with quantification of the phenolic by the chromatographic tests. The Folin-Ciocalteu and UV-Vis methods were useful to instigate us that there were other compounds present in the extract of red propolis in the months with less precipitation. Our questions was confirmed by the LC-Orbitrap-FTMS data and IC_50_ DPPH correlation, which was able to quantify and demonstrate that guttiferones (here considered as phenolic substances) are present in higher concentration in the months of lower precipitation and in turn justify high phenolic in the months of lower precipitation detected in Folin-Ciocalteu method. Pearson’s statistical analysis showed that the general methods for the determination of phenolic compounds and flavonoids showed a greater correlation with each other, whereas the chromatographic methods for the determination of specific flavonoids, in turn, also correlate with each other.

### Antioxidant capacity

All the samples from the seasonal study presented antioxidant activity with values higher than 80.6%, 81.5% and 78.5% in the extract concentration of 80.0 μg/mL for the Propolis A, B and C samples, respectively. The scientific studies by De Mendonça *et al*.^5^ show that Brazilian red propolis presents antioxidant activity with IC_50_ values between 5.0–8.0 μg/mL. However, it was observed high variations in the percentage of antioxidant activity and IC_50_ for propolis A and propolis C samples with IC_50_ values greater than 10–12 μg/mL in the months between March and August. Surprisingly, it was observed that in these months some samples showed maximum concentrations of flavonoids (Month of August for Propolis A and months of July and August propolis C). Propolis A presented IC_50_ of 25.73 μg/mL and 20.40 μg/mL in the months of July and August respectively. Propolis B presented IC_50_ values of 9.25 μg/mL and 8.45 μg/mL for the months of May and July, respectively. Propolis C presented IC_50_ values of 35.47 μg/mL and 51.03 μg/mL for the months of April and June, respectively (Table 1). The Sonoran propolis presented good results of antioxidant activity using DPPH method in concentration range of 12.5 to 100.0 µg/mL^25^. The San Juan Propolis from Argentine presented good antioxidant activity with IC_50_ values between 15.0 to 42.0 μg/mL during a year seasonal cycle^26^.

**Table 1:** Inhibitory Concentration (IC_50_) of the Brazilian red propolis extract against the radical DPPH˙ during the seasonal study (march/2011-february/2012).

Apiary	mar.	apr.	may	jun.	jul.	aug.	sept.	octo.	nov.	dec.	jan.	feb.
Propolis A	10.66	14.82	9.18	15.53	25.73	20.40	2.96	3.45	3.10	2.97	7.68	1.95
Propolis B	8.39	14.81	9.26	6.40	8.45	8.43	7.84	8.26	7.66	7.79	8.51	14.00
Propolis C	12.43	35.47	9.19	51.03	8.71	12.16	10.65	8.04	8.36	6.19	6.44	8.02

**Figure 3:**
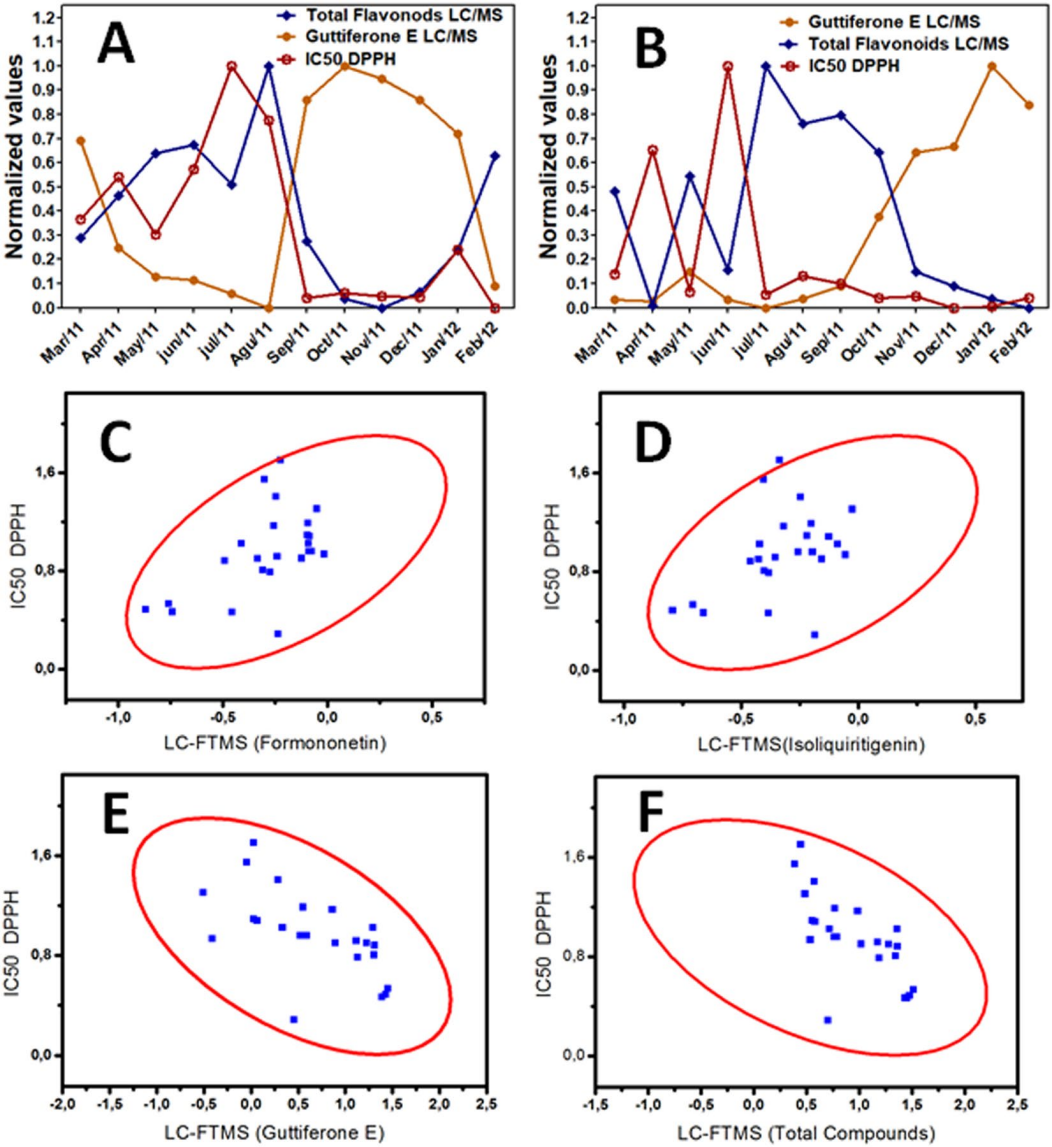
Correlation between IC_50_ values from the DPPH method, flavonoids and guttiferone E concentrations for the Propolis A (**A**) and Propolis C (**B**). Pearson´s correlation between IC_50_ from the DPPH method and formononetin (**C**) with ρ = 0.53, isoliquiritigenin (**D**) with ρ = 0.42, guttiferone E (**E**) with ρ = −0.63 and sum of the total phenolic compounds concentration (**F**) with ρ = −0.69 using concentrations from the LC-Orbitrap-FTMS data.

It was observed that there is low relation of the decrease of the IC_50_ (high antioxidant activity) with values of isolated flavonoid concentration (formononetin, ρ = 0.53; and isoliquiritigenin, ρ = 0.42), but stronger correlation between guttiferone E (ρ = −0.63) determined using LC-Orbitrap-FTMS data. There was a good correlation of the decrease values of IC_50_ with the high concentrations of guttiferones E (ρ = −0.63) and sum of total compounds concentration (ρ = −0.69). The Fig. 3 shows the Pearson´s correlation between some flavonoids (formononetin, isoliquiritigenin), total compounds, guttiferones E, guttiferones B and total guttiferones, and IC_50_ DPPH values. The samples of Propolis A and Propolis C, which presented high IC_50_ values (or decrease antioxidant activity in some months in the raining period), mainly for the months in which there was a decrease in the concentration of sum of total flavonoids and/or guttiferones (the months of March to August in Propolis A; March and June to propolis C).

### Anti-trypanosomal testing and Antibacterial activity

The red propolis samples presented anti-trypanosomal activity during all months including rainy and drought period. The maximum percentage of parasite growing was lower than 3.8% at the concentrations of 10 and 20 µg/mL. The microbiological method showed inhibition of growth of *S. aureus* and *P. aeruginosa* in most months of the seasonal study similar to the results of *trypanosome brucei* (Table 2), except for a few months that coincides with the decrease of the flavonoids/isoflavonoids and guttiferones present in the red propolis. In the months of May and July to has occurred bacterial growth to the extracts of propolis A and in the months of March, April and June for the extracts of red propolis C. The extract of red propolis B showed inhibition of the growth of microorganisms in all months studied. In the months in which the concentration of bioactive substances decreased, propolis A inhibited the growth of *Staphylococcus aureus* ATCC 25293 (118–500 μg/mL) and *Pseudomonas aeruginosa* ATCC 27853 (88–500 μg/mL) with antibacterial activity between 118 and 88 μg/mL, respectively. The same did not occur for extracts of propolis C that showed microbial growth inhibition range for *Staphylococcus aureus* ATCC 25293 (280–500 μg/mL) and *Pseudomonas aeruginosa* ATCC 27853 (210–500 μg/mL) and showing antibacterial activity between 280 μg/mL and 210 μg/mL (see Tables S7 and S8), respectively, and therefore, higher than the other apiaries. The extracts of propolis B inhibited growth over the entire concentration range studied.

**Table 2:** Inhibitory growth (IG%) of *Trypanosoma brucei brucei* S427 of the crude extract of red propolis during the seasonal study (march/2011-february/2012).

Apiary	mar.	apr.	may	jun.	jul.	aug.	sept.	octo.	nov.	dec.	jan.	feb.
Propolis A^a^	0.9	1.9	1.5	1.7	0.1	0.1	0.7	3.7	2.9	3.1	0.1	1.3
Propolis A^b^	0.7	0.7	0.5	0.3	0.6	0.0	0.0	1.9	1.1	0.1	0.0	0.4
Propolis B^a^	1.1	0.9	0.1	0.2	1.9	0.9	0.5	0.0	0.8	0.9	1.7	0.3
Propolis B^b^	2.0	1.1	1.4	1.0	0.2	1.2	0.8	0.1	0.3	0.1	0.5	0.2
Propolis C^a^	1.7	3.8	0.4	3.4	2.3	0.4	0.0	0.3	0.3	3.1	0.5	1.4
Propolis C^b^	0.9	0.6	2.2	0.9	0.1	0.3	1.2	0.7	0.2	1.1	0.8	1.2

^a^Concentration of 20 μg/mL; ^b^Concentration of 10 μg/mL; (IG%) ≤10% is considered Trypomastigotes Activity.

Using the microbiological data from the seasonal study it was possible to establish a range of minimum acceptable inhibitory concentration (<280 µg/mL for the worst months) that could be used more safely and reliably to evaluate the quality of the raw materials and the galenic extracts of red propolis.

**Figure 4:**
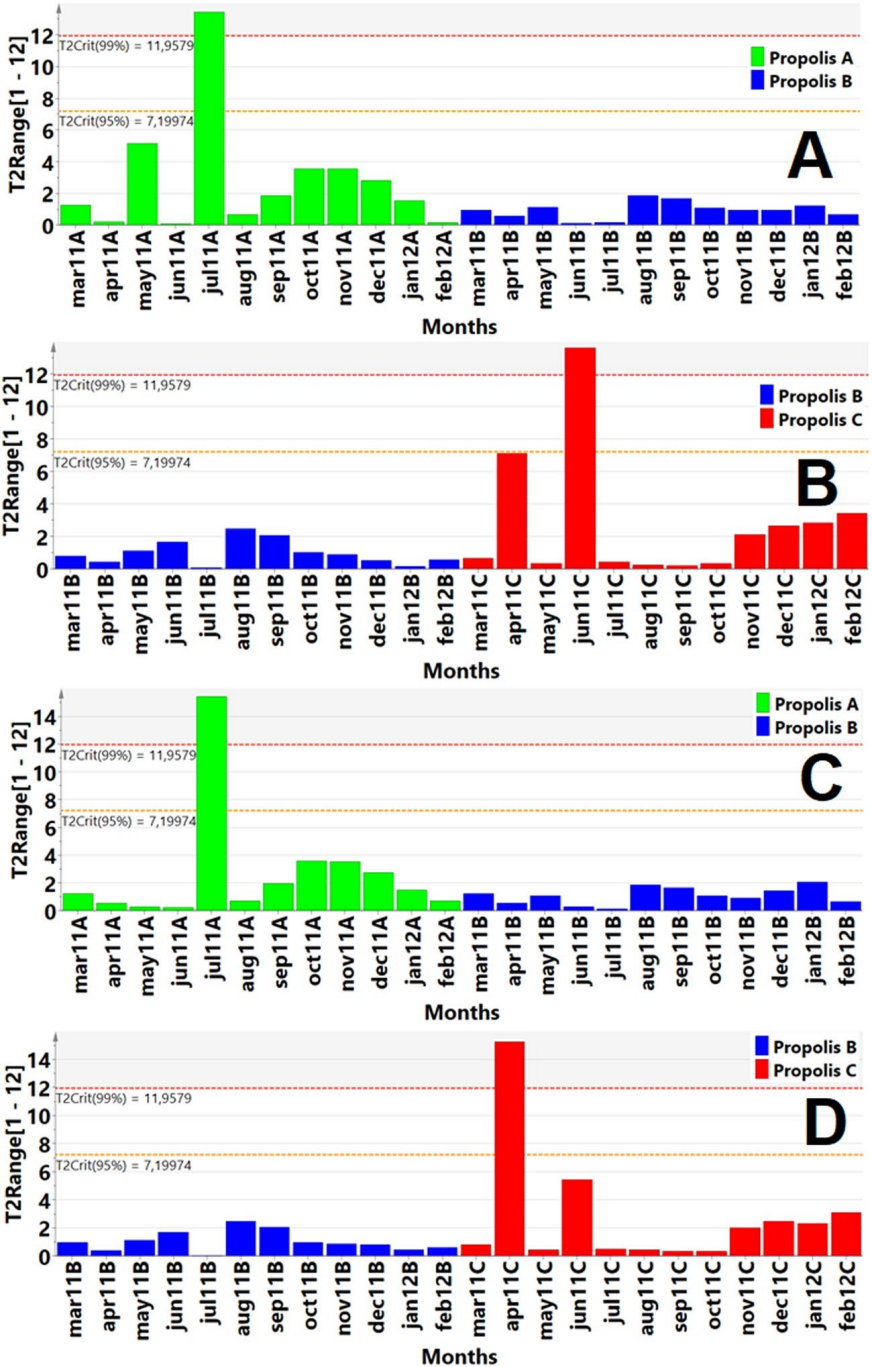
PLS-DA analysis from the interaction between all components (Component 1, all months of the seasonal study as primary variable), (Components 2 to 5, phenolic compounds concentrations and Components 6 to 12, antibacterial activity concentrations tested as independent variables) were analysed using *S. aureus* (**A,B**) and *P. aeruginosa* (**C,D**). (**A**) Paired PLS-DA Hotelling´s T2 test compares propolis A (green) with propolis B (blue) at significant levels 95% (T2: 7.19) and 99%(T2: 11.95) as upper critical limits (UCL) in the antibacterial test against *Staphylococcus aureus* resulting in outliers in propolis A (July sample). The PLS-DA model was validated by CV-ANOVA, p = 0.0005, F = 8.16. (**B**) Paired PLS-DA Hotelling´s T2 test compares propolis B (blue) with propolis C (red) at significant levels 95% (T2: 7.19) and 99% (T2: 11.95) as upper critical limits (UCL) in the antibacterial test against *Staphylococcus aureus* resulting in outliers in propolis C (June sample). The PLS-DA model was validated by CV-ANOVA, p = 0.0199, F = 3.78. (**C**) Paired PLS-DA Hotelling´s T2 test compares propolis A (green) with propolis B (blue) at significant levels 95% (T2: 7.19) and 99% (T2: 11.95) as upper critical limits (UCL) in the antibacterial test against *Pseudomonas aeruginosa* resulting in outliers in propolis A (July sample). The PLS-DA model was validated by CV-ANOVA, p = 0.0002, F = 9.20. (**D**) Paired PLS-DA Hotelling´s T2 test compares propolis B (blue) with propolis C (red) at significant levels 95% (T2: 7.19) and 99% (T2: 11.95) as upper critical limits (UCL) in the antibacterial test against *Pseudomonas aeruginosa* resulting in outliers in propolis C (April sample). The PLS-DA model was validated by CV-ANOVA, p = 0.0104, F = 4.44.

The Hotelling T^2^ paired test, showed an inverse correlation between the low concentrations of phenolic compounds (the data were extracted from the LC-Orbitrap-FTMS) and bacterial growing in high concentrations tested. The months in which the flavonoid and guttiferones concentrations were low, there were bacterial growing and it was detected in upper critical levels of Hotelling T^2^ paired test (significance levels of 95% and 99%) from PLS-DA graphs and corresponds to higher values of bacterial growing to the Propolis A and Propolis C (Fig. 4; see Figs. S7 and S8). In the months in which formononetin concentrations are low and the absence of concentrations of guttiferones has occurred, it was observed the presence of samples in outliers on the Hotelling T^2^ paired test (Fig. 4) and PCA-X graphs (see Figs. S7 and S8). The PCA-X graphs showed higher presence of outliers to the formononetin isoflavonoids (4 outliers) in relation to isoliquiritigenin (2 outliers), sum of total flavonoids areas (2 outliers) and guttiferone E (3 outliers) during anti-staphylococcal activity (see Figs. S7 and S8).

**Figure 5:**
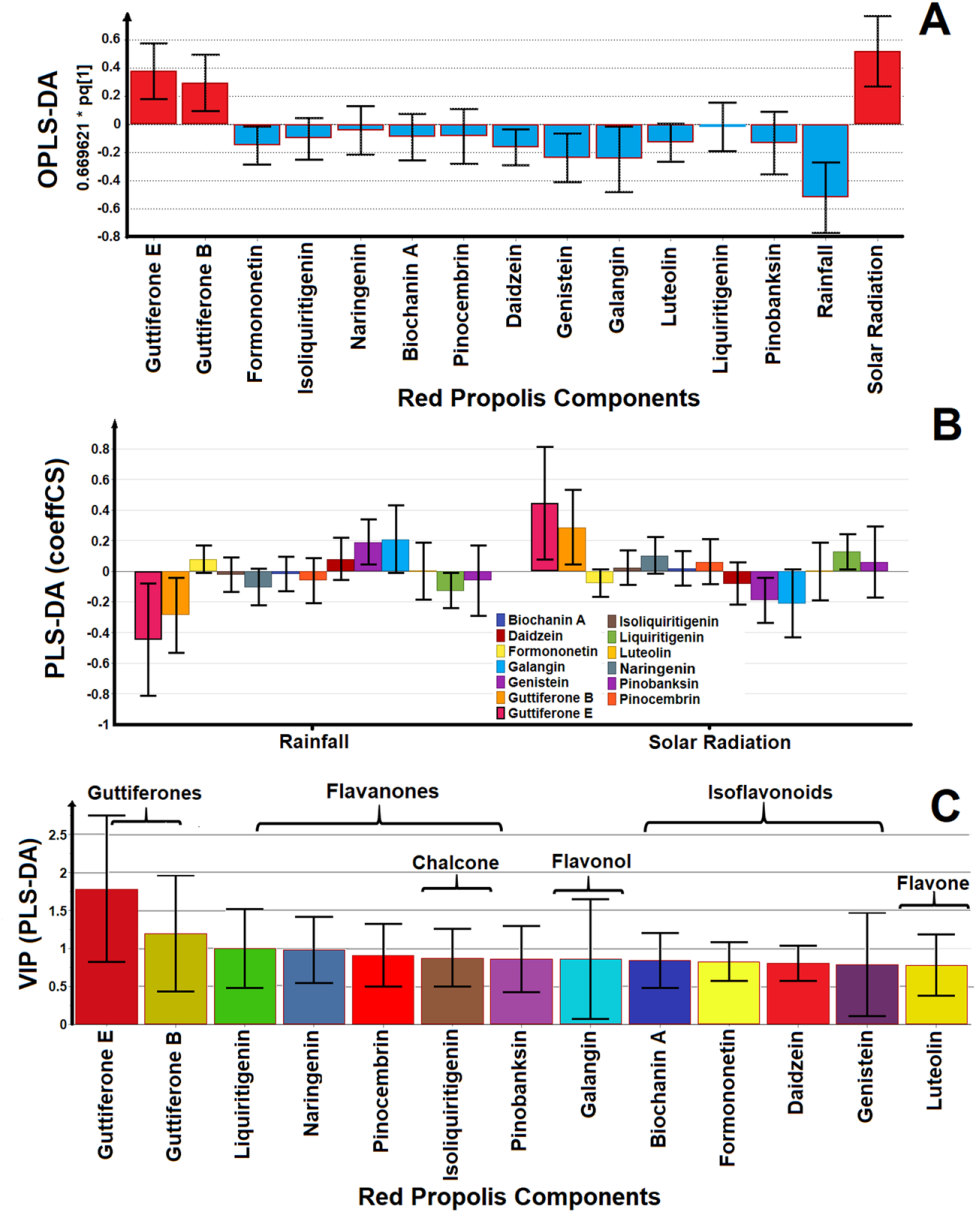
Multivariate statistical analysis distinguished the phenolic compounds on the antibacterial activity against *Staphylococcus aureus*. (**A**) OPLS-DA and (**B**) PLS-DA analysis during the rainfall and drought periods. (**C**) VIP analysis (Variable Importance in Projection) extracted from the PLS-DA plot identified principal phenolic compounds (guttiferones) and classes of flavonoids (flavanones) responsible by anti-staphylococcal activity during the seasonal study. The OPLS-DA model was validated by CV-ANOVA, p = 0.000004 (p < 0.05), F = 12.47. The PLS-DA model was validated by CV-ANOVA, p = 0.000003 (p < 0.05), F = 12.90.

Orthogonal PLS analysis showed a greater tendency of flavonoids to correlate positively in the rainy season, except for liquiritigenin. However, guttiferones present a greater tendency to correlate in the period of intense sun, in other words, the flavonoids tends to inhibit growth of microorganisms in the period of intense rains and the guttiferones exert the same function in the period of prolonged droughts. Among the flavonoids, galangin, genistein, daidzein, formononetin are strongly discriminated in rainy periods and other flavonoids are reasonably discriminated as Biochanin A, pinocembrin, naringenin, pinobanksin and isoliquiritigenin (Fig. 5A). Discriminant analysis using the PLS-DA test shows the flavonoids (galangin, genistein, daidzein, formononetin) correlating strongly with the rainy months, some flavonoids are indifferent in the intense solar radiation period (isoliquiritigenin, Biochanin A and Luteolin). Other flavonoids tend to correlate in the period of intense sun (liquiritigenin, naringenin, pinobanksin and pinocembrin). The guttiferones presented a positive correlation with a period of intense sun (Fig. 5B). Discriminant analysis of the rainy months and months of intense solar radiation was inserted in the PLS-DA analysis and this showed a greater discriminatory power in relation to the OPLS-DA analysis. In this way, we tested the Variables Important in the Projection (VIP) analysis, a variant mode from the (PLS-DA) test to verify which flavonoids present in the red propolis extract are contributing strongly to the antimicrobial activity in this complex mixture (values above 0.5)^38^. The VIP analysis showed that guttiferones, liquiritigenin and naringenin presented values above 1 being, therefore, these flavonoids are strongly contribute with antimicrobial activity. However, other flavonoids present in the red propolis extract presented values above 0.5 and therefore they are also considered important and may contribute to the antimicrobial activity of the extracts of red propolis, especially at times with low concentration of guttiferones, the flavonoids assuming an important role in the synergy of the effect biological (Fig. 5C). The OPLS-DA and PLS-DA data observed other flavonoids; galangin and genistein; presented as important substances. Indeed, galangin and genistein were observed as discriminate components in the rainy season and VIP analysis demonstrated great variation and can also contribute with the antibacterial activity in rainy period (Fig. 5C). The VIP analysis has discriminated the compounds of major antimicrobial importance taking into account their respective concentrations (normalized), as well as related these concentrations with the months that occurred growth of the tested microorganism and the months that did not occur grow. Thus, in the months that the highest growth of microorganism occurred, the concentration of guttiferones was very low, but other substances such as formononetin and isoliquiritigenin were relatively high and in this way theses flavonoids should assume important role (important weight) in inhibiting the growth of *Staphylococcus aureus*, but failed. In the months of intense sun the concentration of guttiferones was high and no microorganism presented growth. So in this way, the guttiferones received important role (important weight) in the inhibition of growth. This is justify the flavonoids of higher concentration (formononetin and isoliquiritigenin) were not important in the VIP analysis, while other flavonoids with less concentration (galangin, genistein, daidzein) had an important role in the VIP test.

## Discussion

Several factors (Biotic and Abiotic) can directly influence the biosynthesis of plants visited by bees and consequently the composition of the propolis studied. The abiotic factors are cited (light intensity, temperature range and average temperature, humidity, wind speed, solar radiation, water availability, rainfall) and biotic factors as (habit, cultivation site, season, age of plant, competition with neighboring plants, interaction with insects and other pathogens), can directly affecting the concentration of flavonoids, phenolic acids and guttiferones^39^. In general, these compounds are secondary metabolites from plants and the secondary metabolites biosynthesis in plants is regulated in space and time^40^ and is affected by abiotic environmental factors, such as light intensity, soil minerals, osmotic stresses (drought and salinity), and seasonality^41,42^. Seasonal variation was identified in hypericin content of *Hypericum peroratum* (St. John´s Wort) and in two consecutive seasons, the hypericin/pseudohypericin concentrations in a broad leaf biotype varied from a winter minimum of less than 100 ppm to a summer maximum approaching 3000 ppm^43^. The authors, Paneitz & Westendorf^44^, demonstrated that emodin levels in plants depend on season and light intensity and highest amount of total anthraquinones (where approx. 50% was emodin) in the leaves of *Rheum undulatum* in Europe was observed in springtime (April) followed by a continuous decrease during the summer with the lowest anthraquinone amounts in late summer (September).

Authors as Davis & Schwinn^45^, consider the secondary metabolites pathway for the flavonoids and isoflavonoids, as a mixed metabolic pathway between: (1) the shikimic acid and arogenate pathways and (2) the acetate/malonate pathway. Many authors as Wu *et al*.^46^ and Cuesta-Rubio *et al*.^47^ also consider the polyprenylated benzophenones secondary metabolites pathway from shikimic acid starting in L-phenylalanine as precursor of 2,4,6 trihydroxibenzophenone catalysed by the benzophenone synthase enzyme. This second metabolic pathway (acetate/malonate) in turn is also responsible for the addition processes of the isoprenyl and geranyl groups with help of transferase-enzyme addition the prenyl (geranyl) pyrophosphate to the trihydroxibenzophenone.

In the rainy and drought seasons, some modification in the plant habitat occur like as high water availability, high relative humidity, high solar incidence, pathogens incidence in the plants (fungal, bacteria) and insect (bee)-attack to plant due to low-food offer. A mechanism of upregulation occurs due to the interactions insect (bees)-plants responsible by the red propolis composition favouring the increase of flavonoids concentration. Some interactions bees-plants promote the called stress-induced phenylpropanoids metabolism as proposed by Dixon and Paiva (1995)^48^. According to Dixon and Paiva (1995)^48^, this stress condition can induce the phenylpropanoids metabolism in the plants to produce in great amount the secondary metabolites flavonoids, isoflavonoids, guttiferones and phenolic compounds by plants.

During the drought period, the acetate-malonate pathway is more favourable in relation to the shikimic acid pathway, but in equilibrate condition, leading to a higher production of polyprenylated benzophenones as guttiferones and xanthochymol by the plant. The higher solar radiation incidence can be favourable to a higher photosynthesis, plant hydric-stress and consequently this fact occurred favouring the higher polyprenylated benzophenones biosynthesis. The biosynthesis of benzophenones is not fully elucidated yet, being considered of multiple pathways according to Cuesta-Rubio *et al*. and Baggett *et al*.^47,49^. The propolis production by bee is only a response from natural climate variation from plant during the cycles of rainy and drought periods.

Another possibility to be explored can be the fact that the bees prefer to collect resins, sap and substrates for the elaboration of the red propolis of a certain group of plants in the period of intense rains, prevailing the group of flavonoids and isoflavonoids like as *Dalbergia ecastophylum* plant from the Fabaceae family. But in the intense summer, the bees have a preference for collecting substrates in another particular group of guttiferone-rich plants as *Garcinia xanthochymus* from Clusiaceae family^49^. The research of Kato *et al*.^50^ on the plant-pollinator interaction in tropical monsoon forests in Southeast Asia has shown that bees are responsible for 55% of plant pollination. In addition, 4 bee species (including Apis cerana), 2 small bee species, 2 butterflies species and 2 beetle species are the main responsible for the process of pollination of the species *Dalbergia rimosa* Roxb. The research of Crepet & Nixon^51^ also demonstrated close relationship with melliponine and other highly derived bee pollinators from the fossil Clusiaceae flowers data.

The IC_50_ data from the antioxidant activity assays, Pearson´s correlation and Fig. 4 data demonstrate that total flavonoid concentrations are not solely responsible for the antioxidant action of red propolis extracts. A synergistic effect of flavonoids and guttiferones is being observed and promotes the decrease of IC_50_ values (greater antioxidant activity of red propolis extracts) between 1.95 and 7.67 μg/mL (Propolis A), 6.4 and 8.51 μg/mL (Propolis B) and 6.20 and 8.36 μg/mL (Propolis C). Some polyprenylated benzophenones, xanthochymol, isoxanthochymol, guttiferones E, aristophenones A and B presented antioxidant activity in the range of IC_50_ 73–125 µM^50,52^. In addition, garcinol and isogarcinol presented antioxidant activity at the concentration of 10 and 13 µM^49,53^. Some tetrahydroxi-methoxi-benzophenones presented antioxidant activity at the range 0 f 3.6–6.6 µM on the human Low-Density Liporpotein (LDL) oxidation assay^54^.

The LC-Orbitrap-FTMS method detected and quantified guttiferones and several phenolic compounds including flavonoids and isoflavonoids present in the red propolis from Alagoas-Brazil, but the others quantification methods of total phenols content like as Folin-Ciocalteu, UV-vis and LC-DAD-UV were effective in to detect seasonal variations minacious in red propolis extract. The Pearson´s statistical analysis demonstrated seasonal correlation between total flavonoids and guttiferones concentrations with meteorological data special for the increasing of flavonoids in rainfall season and guttiferones with intense solar radiation in the intense summer.

The antioxidant and anti-bacterial assays demonstrated variations on the red propolis raw-material quality, but anti-trypanossomal test did not detect this seasonal variation. Our preliminary studies demonstrate that the Brazilian red propolis from the region of Alagoas collected in the period 2011–2012 presented a trypanocide activity in the concentration range between 10 and 20 μg/mL. Some *in vitro* assays have demonstrated the trypanocide activity of red propolis against Trypanosoma strains. Regueira Neto *et al*.^55^ demonstrated growth inhibition against *Trypanosoma cruzi* with IC_50_ values < 31 μg/mL for red propolis samples from Pernambuco, Brazil. Gressler *et al*.^56^ demonstrated trypanocide activity of propolis extract against *Trypanosoma evansi* at the concentrations range of 5 to 10 μg/mL. Salomão *et al*.^57^ demonstrated activity of Brazilian green propolis against *Trypanosoma cruzi* with an IC_50_ value of 8.5 μg/mL. Phenolic compounds isolated from the Nigerian red propolis extract inhibited the growth of *Trypanosoma brucei* S427WT with IC_50_ values of 10.0 μg/mL (calycosin), 4.1 μg/mL (8-prenyl naringenin), 7.8 μg/mL (macarangin), 8.3 μg/mL (vestitol), 4.2 μg/mL for the Nigerian propolis extract^58^. Other isolated phenolic compounds showed trypanocide activity like as ascofuranone (IC_100_ = 0.03 μM with glycerol), considered potent and specific inhibitor of the glycerol −3-phosphate-dependent mitochondrial oxygen consumption of *T. b. brucei* bloodstream forms^59^. Two flavonols, 7,8-dihydroxyflavone and quercetagetin, were trypanocide at the submicromolar concentrations (IC_50_ = 0.16 and 0.8 μM), in addition to cisampeloflavone (IC_50_ = 1 μM), curcumin (IC_50_ = 0.83 μM) and justicidin B (IC_50_ = 0.55 μM) inhibited growth of *Trypanosome brucei rhodesiense*^59^.

The DPPH method and antibacterial activity (microbiology test) have shown to be sensitive to detect variations in the concentrations of phenolic compounds present in red propolis extracts, thus they also are important tools for monitoring the quality of extracts of Brazilian red propolis. The Pearson´s statistical analysis demonstrate correlation between IC_50_ values from DPPH test, with synergistic effect between guttiferones and the sum of total phenolic compounds. The relationship between the climate and phenolic compounds was demonstrate and the variation of these compounds proven to influence on the biological activity of this important Brazilian apiceutical raw material. The results of the seasonal monitoring using minimum inhibitory concentration demonstrated that the MIC test presents a greater sensitivity to discriminate samples of red propolis with biological activity. The higher microbial bioburden (1 × 10^6^ CFU/mL) used in microbiological assays compared to the trypanosomicidal test (2–3 × 10^4^ trypanossoma/mL) may be one of the reasons why it makes the assay more sensitive and discriminant.

According to Bueno-Silva *et al*.^28^, the MIC values through a year analysis for *S. mutans* varied from 62.5–125 to 125–250 µg/mL and for *S. aureus* varied from 31.2–62.5 to 125–250 µg/mL and the MBC through a year analysis varied from 250–500 to 500–1000 µg/mL for *S. mutans* and from 62.5–125 to 125–250 µg/mL for *S. aureus* and isoliquiritigenin, vestitol, neovestitol and synergism of phenolic compounds are responsible to the antibacterial properties. The authors Regueira Neto *et al*.^29^, compared the antibacterial activity of red propolis samples from Pernambuco State during rainy season (April to August 2014) and drier season (December/2015 to March/2016), then showed different MICs values for *Staphylococcus aureus* and *Escherichia coli*. The rainy season presented lower MIC values for these strains than dryer season. Regueira-Neto *et al*.^29^ studies showed that the concentrations of quercetin, caffeic acid and ellagic acid were approximately 2 times higher in the dry season compared to the rainy season, while apigenin and luteolin were higher in the rainy season. The variations in flavonoid concentrations of red propolis samples from Pernambuco-Brazil can be explained by the low amount of rainfall in the dry season and also in the rainy season. The mean of rainfall intensity was lower than 50 mm, and the rainfall-accumulated amount was 222 mm per month during rainy season and in dry season the mean rainfall intensity was lower than 25 mm and the rainfall-accumulated amount of 152 mm per month (see Fig. S9). The National Institute of Meteorology^35^ data from the Brazilian government show that even in the rainy season the amount of rainfall was regular and in the sample collection period (April/2014 to August/2014) the months of April and May exceeded 250 mm of rainfall-acumulated amount and amount superior to 100 mm in the other collected months (see Fig. S9). This may also be one of the reasons to the low variations of flavonoids in the rainy period for red propolis samples from Pernambuco-Brazil. In addition, the Seasonal collection period (2015–2016 cycle) of the Regueira-Neto *et al*.^29^ research coincides with the El Niño phenomenon^36^ **(**see Fig. S5**)**, which causes decreasing in intensity and frequency of rainfall in the north eastern region of Brazil during the rainy period (see Fig. S9).

The Bueno-Silva *et al*.^28^ research, despite not reporting the period of seasonal collection of the samples, states that during the period of intense rainfall and higher relative humidity the amount of isoflavonoids vestitol and neovestitol were intense with better MIC values for the bacteria studied. The data from Bueno-Silva *et al*.^28^ have corroborated our period of intense rainfall (rainfall >100 mm) from late April to early September and higher concentration of isoflavonoids and flavonoids. However, our best antibacterial activities occurred in the dry period when the concentration of guttiferones are very intense. Argentinian propolis showed that the extracts of propolis collected in the summer and spring had a higher content of phenolic and flavonoid contents than the samples collected in winter and autumn^56^. The propolis collected in summer and autumn showed higher antibacterial activity (30 μg/mL) than the other samples (MIC values between 30 and 120 μg/mL)^60^.

The PCA-X and Hotelling T2 paired test analysis were only used to demonstrate the absence or decrease of the concentration of some constituents may result in the outliers (months in outliers) (Fig. 4; see Figs. S5 and S6). The PCA and Hotelling T2 test analysis were not to faithfully attribute a direct structure-activity relationship between flavonoids and minimum inhibitory concentrations, but these analysis were important to start the investigation the phenolic compounds responsible by the antibacterial activity. It was necessary the use OPLS-DA, PLS-DA and VIP analysis to discriminate the principal components in red propolis extract with antibacterial activity. The study was conducted using the *Staphylococcus aureus* due to the high bacteria growth during the antibacterial activity test using the seasonal samples. The VIP (PLS-DA) analysis of the phenolic compounds quantified by chromatographic techniques showed that guttiferones E and B were the most important components of red propolis followed by flavonoids liquiritigenin and naringenin (VIP value > 1), but VIP values between 0.5 and 1 demonstrate no less important components (Fig. 5). The fact that the extracts of red propolis are showing good antimicrobial activity can be explained by the high concentration of guttiferones, liquiritigenin, naringenin and because the present flavonoids (isoliquiritigenin, galangin, genistein, other isoflavones and flavones) also promote adequate antimicrobial activity^23^.

Polyisoprenylated guttiferones exert potent antimicrobial, antiviral and tumor cells activities. The guttiferone A was cited in scientific literature with antibacterial activity^61^. Iinuma *et al*.^62^ demonstrated that benzophenones from Garcinia exhibit antibacterial activity with low inhibitory concentration against MRSA (Methicillin-Resistant *Staphylococcus aureus*) strains and, in particular, xanthochymol showed MICs between 3.1 and 12.5 µg/mL and some benzophenones has reported antibacterial activity against gram-negative and gram-positive^62,63^. Lenta *et al*.^64^ demonstrated antimicrobial activity of Simphonin, a prenylated xanthone. Almeida *et al*.^65^ demonstrated antibacterial activity of 7-epiclausianone, a polyisoprenylated benzophenone, with MIC between 1.25 and 2.5 ug/mL. Cunha *et al*.^66^ demonstrated anti-biofilm properties of ent-Nemerosone against *Streptococcus mutans*. The report of Seeber *et al*.^67^ demonstrated that isomers of the clausionones (polyisoprenylated benzophenones) obtained in the Caribbean region presented cytostatic and virostatic activities to act as an inhibitor of the topoisomerases, telomerases and as regulator of the MAPK signal transduction inhibited in various types of cancer.

The flavonones, naringenin and pinocembrin, inhibit the growth of gram-positive bacteria including *S. aureus* in concentrations higher than 4 µg/mL^68^. The flavanones with C-5, C-7, C-2´, C-4´ and C-6´ hydroxylation and substituted by prenyl groups at 6-C or 8-C positions both showed activity against MRSA *S. aureus*^69^. Flavonoids as naringenin and taxifolin have to attenuate quorum production sensing (Q-S)-controlled virulence factors in *P. aeruginosa*. Naringenin, at suitable concentrations, may alter the membrane fluidity of bacteria, as well as inhibit the expression of several Q-S controlled genes of *P. aeruginosa* and consequently reducing the virulence of pathogenic bacteria^70^. Studies of Gaur *et al*.^71^ have shown minimal inhibitory concentration of liquiritigenin (MIC 50 µg/mL) for four MRSA strains and isoliquiritigenin showed MIC of 50 µg/mL for three MRSA strains. In another study, liquiritigenin and isoliquiritigenin were able to inhibit growth of *Mycobacterium tuberculosis* at a concentration of 25 µg/mL^72^. Liquiritigenin, one of the most significant active components of licorice decreasing the production of α-hemolysin and can prevent human lung cells (A549) from α-hemolysin-mediated injury^73^. Sechet *et al*.^74^ demonstrated that isoliquiritigenin showed immunoregulatory properties by stimulating colonic epithelial cells to produce Human Beta-Defensin-3 (HBD3) against pathogenic bacteria in addition to acting synergistically with antimicrobial peptides (HBD3) in cells of the intestinal mucosa in infectious treatments and dysbiosis disease in humans.

The flavonols, Galangin and galangin-3-methyl ether, are considered lipophilic compounds and have antibacterial activity with MIC between 1 and 0.25 μg/mL against *S. aureus* and gram-positive bacteria^68^. Cushine *et al*.^75^ demonstrated that galangin inhibits growth of *S. aureus* strain resistant to the antibiotic 4-quinolone by inhibiting enzyme to IV topoisomerase during DNA synthesis.

Isoflavones (genistein, biochanin A, formononetin and daidzein) have shown moderate antibacterial activity against *S. aureus* with MIC values >128 μg/mL for genistein^76^. The research performed by Neves *et al*.^77^ demonstrated a formononetin isolated from red propolis extract presented antibacterial activity against *Staphylococcus aureus* (MIC 200 μg/mL), *Pseudomonas aeruginosa* (MIC 200 μg/mL) and antifungal activity against candida sp. (MIC 25 μg/mL). Prenyl group in C-6 and C-8 position present in isoflavones increase the antibacterial activity of genistein with MIC values of 16 μg/mL. Mukne *et al*.^78^ states that isoflavones have action against gram-positive bacteria and anti-staphylococcal action and that genistein can inhibit bacterial growth by inhibition of topoisomerase IV. Wang *et al*.^79^ demonstrated that genistein has been acting in the NorA efflux of *S. aureus* bacteria.

**Figure 6:**
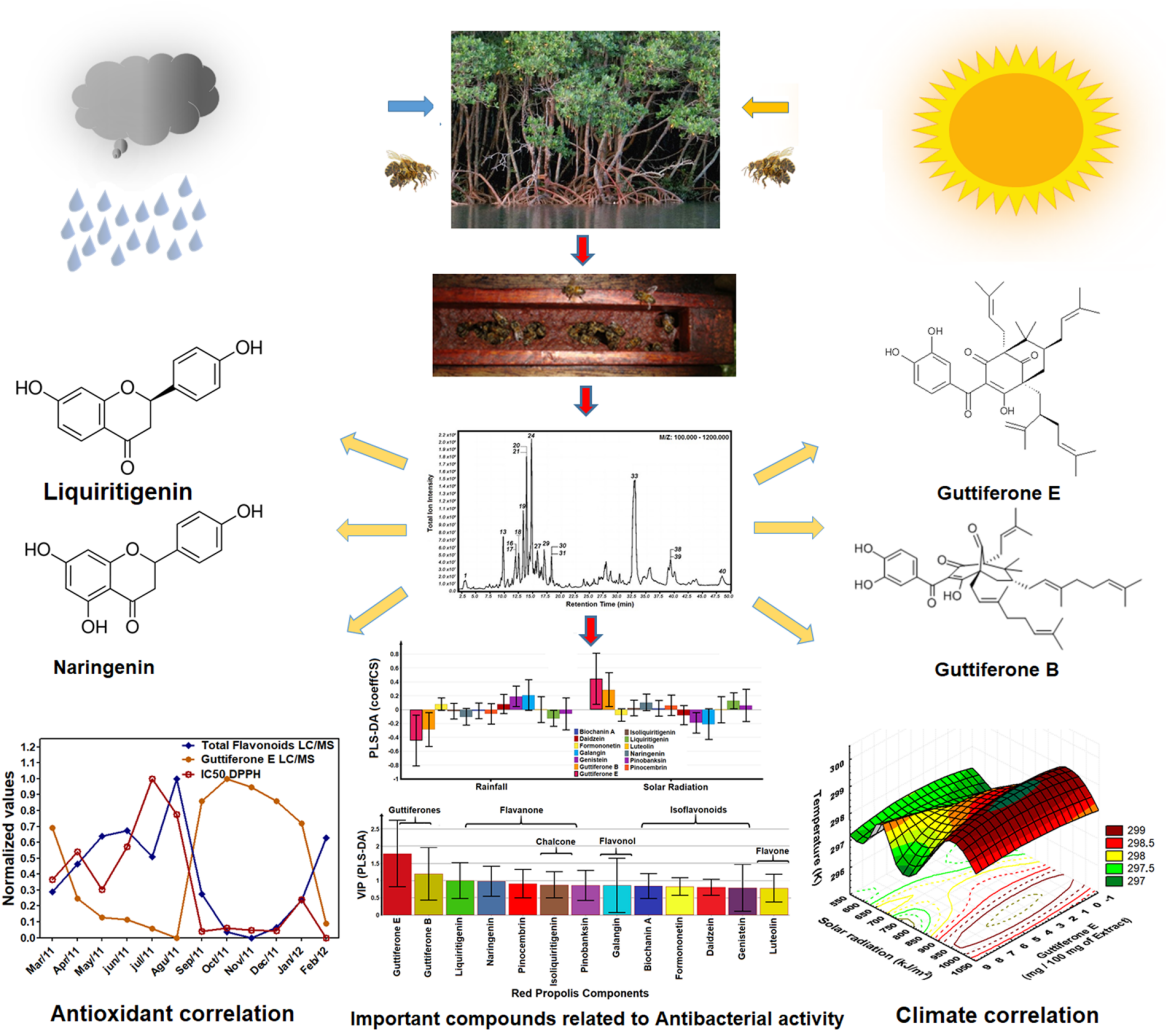
The climate effect and its chemical and biological correlations during a one-year cycle seasonal study of the Brazilian red propolis extract. The variation of the polyphenols (flavonoids and polyprenylated benzophenones) were detected using LC-ESI-Orbitrap-FTMS, HPLC-DAD-UV, UV-vis and Folin-Ciocalteu methods.

The presence of certain functional groups (phenolic hydroxyl, prenyl, geranyl) in flavonoids at specific positions may increase anti-staphylococcal activity against MRSA strains^80–82^. The VIP analysis has shown the presence of polyisoprenylated substances (guttiferones E and B) and flavonoids with phenolic hydroxyls at positions C-5, C-7 and C-4’ (naringenin), C-7 and C-4´ (liquiritigenin) have been shown to be responsible for the antibacterial activity of the extracts of red propolis in this study of seasonality. The VIP analysis also showed that all compounds assume values above 0.5 and therefore are no less important. So, we can not rule out the possibility that other flavonoids not quantified in this study and the possibility of synergistic effect with other classes of compounds such as isoflavans^31^ (vestitol and neovestitol), phenolic acids, and pterocarpans^83^ (medicarpin), not monitored during a seasonal study^34^. The red propolis extract can be understand as a multi-constituent mixture formed by several flavonoids, aromatic, phenolic, terpenes and guttiferones with multi-targets purpose^84^. The antibacterial activity could not attribute only one substance, but rather to the combination of compounds actuating in synergistic effect and resulting in its antibacterial activity^23,84^. The Fig. 6 summarized this seasonality study from Brazilian red propolis extract.

## Experimental

### Authorization document and collection of the red propolis

This research was previously authorized by regulatory agencies for control of Brazilian Genetic Heritage and biodiversity conservation for collection and transportation (Document number of authorization 010124/2012-8 and can be accessed in http://www.cnpq.br/documents/10157/e7fec3ea-f4cd-4832-9cc0-4adcf789ab51).

The BRP raw materials (30 g) were collected between months March of 2011 and February of 2012 in three apiaries. The Ilha do Porto apiary (Propolis A) with geographical coordinates of south latitude: 9° 44.555´, west latitude: 35° 52.080´ and height of 18.1 meters. Primavera apiary (Propolis B) with geographical coordinates of south latitude: 9° 42.258´, west latitude: 35° 54.391´ and height of 35.5 meters and Paripueira apiary (Propolis C) with geographical coordinates of south latitude: 9° 26.448´ west latitude: 35° 31.710´ and height of 4.7 meters. The meteorological data of rainfall intensity, solar radiation, humidity, temperature, pressure, wind speed were acquired at the National Institute of Meteorology from the Brazilian government site (www.inmet.gov.br)^35^ in order to correlate the chemical data and biological with meteorological data.

### Sample preparation

The samples were collected free from animals killed and others materials. Ten grams (10 g) of raw material of propolis were used to extract the active constituents using a maceration method with 80% ethanol (150 mL) assisted by manual agitation in the first 24 hours allowing the samples in stand for 72 hours before the collect the first extract. The limpid supernatant liquid was removed with a pipette and the resinous mass deposited at the deep of glass flask was submitted to new extraction with more 150 mL of 80% ethanol. At the end of 2 cycles of extraction, a rotary evaporator (Fisatom, Brazil) concentrated the extracts and about 5 to 7 grams of crude extract of propolis was obtained (36 samples)^24,85^. The dry mass was stored in a freezer at −20 °C until further analysis. A mass of 100 mg was exactly weighed, solubilized with ethanol, diluted and submitted to method validation and quantification in four different methods: Folin-Ciocalteau, UV-vis, LC-DAD and LC-Orbitrap-FTMS methods. Furthermore, antioxidant activity using DPPH method and biological activities were monitored by microbiological method (MIC assay) using *Staphylococcus aureus* ATCC 25293 and *Pseudomonas aeruginosa* ATCC 27853 and *trypomastigotes* activity method using *Trypanosoma brucei brucei S0247*.

### Antioxidant capacity

The seasonality samples (Extracts of Propolis A, Propolis B and Propolis C) were prepared at an initial concentration of 1.0 mg/mL (stock solutions) in the absolute ethanol. An aliquot of 400, 180, 60, 30 and 10 μL was transferred to a volumetric flask of 5 mL, and then 2.0 mL of DPPH solution (3 mM) was added and diluted with absolute ethanol until achieving final concentrations of 80, 36, 12, 6, 3 and 1 μg/mL. The reaction was left to develop in the dark at room temperature (25 °C) over 30 min. The absorbance readings were then performed with a spectrophotometer (Model UV-1240, Shimadzu, Kyoto, Japan) at 518 nm^86^.

### *In vitro* anti-trypanosomal test

Trypanosoma brucei brucei S427 (blood stream form) and the concentration of parasite 2–3 × 10^4^ trypanosomes/mL was used for activity test of red propolis. The crude extracts of the three samples of seasonal study were subjected to testing for anti-trypanosome activity. The crude extracts were exactly weighted (10.0 mg/mL) in DMSO. Suramin (Sigma-Aldrich) as positive control and was prepared using a 10 µM (1 in 100 dilution (HMI-9 medium) serially dilute 1:1 (in dilution template) i.e. 10, 5, 2.5, 1.25, 0.625 µM to 0.08 µM. To give assay positive control range of 1.000 to 0.008 µM. A amount of 200 µg/mL test solutions were prepared in column 2 by pipetting 4 µL of (10 mg/ml) test stock solution and adding 196 µL HMI-9 medium (Invitrogen) into each well. A volume of 100 µL HMI-9 medium was pipetted into the wells and serial 1:1 dilutions are carried out using a multi-channel pipette. Add 100 µL of trypanosomes to each well and incubate the plate at 37 °C, 5% CO_2_ with a humidified atmosphere for 48 hours. After incubating for 48 hours add 20 µL of Alamar blue was incubate under the previous conditions for a further 24 hours. The concentration was determinate using fluorescence method with the Wallac Victor 2 microplate reader. The wavelength of excitation (530 nm) and emission (590 nm) was adjusted for quantification. The anti-trypanosome was expressed as the mean result in triplicate.

### Antibacterial assay

The antibacterial test was carried out using broth microdilution assay into microplates (96 wells) containing 100 μL/well of Muller Hinton broth, following the procedure described by CLSI^87^, with some modifications. A stock solution prepared at 10 mg/mL using ethanol 96 °GL was diluted with phosphate buffer (pH 7.4) in Muller Hinton broth. Serial dilutions (in the ratio of 3:4) were prepared in concentrations ranging from 66 to 500 µg/mL, in microplates. About 30 μL of bacterial suspension (about 1.5 × 10^8^ CFU/mL) was added to wells containing 100 μL of Muller Hinton broth medium with different final concentrations of propolis extract. The results were observed after addition of 40 µL of resazurin solution (100 μg/mL) and re-incubation at 36 °C up to 2 hours. Blue spots in microplate show absence of *S. aureus* and *P. aeruginosa* growing and pink spots in microplate show bacterial growth. Serial dilution of ethanol at 96 °GL in Muller Hinton broth without bacterial streams was performed as negative control and serial dilution of ethanol at 96 °GL in Muller Hinton broth with bacterial streams was performed as positive control. The test was assayed in triplicate^34^.

### Statistical analysis and data correlation using multivariate analysis

The assays of determination of total flavonoids using UV-vis, total phenols content using Folin-Ciocalteu and antioxidant activity were done in triplicate and expressed graphically by the average and standard deviation using the software Graphpad Prisma, version 5.0. The assays of the individual flavonoids determination using (LC-DAD-UV and LC-Orbitrap-FTMS methods) and the guttiferones determination using (LC-Orbitrap-FTMS) were also expressed graphically by average and standard deviation of duplicate assay using the same graphpad Prisma software version 5.0.

The multivariate analysis was established between flavonoids concentrations and, guttiferones concentration and meteorological data (Relative Humidity/%, Rainfall intensity/mm, Solar radiation/kJm^2^, temperature/K) obtained from the National Institute of Meteorology (INMET.gov.br) through surface graphs using Statistica software, version 13.0.

The multivariate comparative correlations between chemical, UV-vis and chromatographic methods were expressed using Matrix Scatter graphs and, the Pearson´s correlation using Software ORIGIN 8.0 version was applied. The same method was used to establish multivariate correlation between LC-Orbitrap-FTMS data (isoliquiritigenin, formononetin, sum of total flavonoids, guttiferone E in isomeric mixture concentrations) and IC_50_ of the DPPH method for all months of the seasonality study of the Propolis A and Propolis C^88^.

The correlations analysis between the phenolic compounds variations on the months with microorganism (*Staphylococcus aureus* or *Pseudomonas aeruginosa* strains) growth were established using PCA-X and Hotelling T^2^ paired statistical test through SIMCA P software, version 12.0^15,89^. A matrix 36 × 12 (36 samples of the seasonality of propolis A, propolis B, propolis C and 12 important variables of the seasonality study using LC-Orbitrap-FTMS data was done. The important variables were defined as: 36 samples of seasonality (primary component), concentration of isoliquiritigenin (component 2), concentration of formononetin (component 3), concentration of the sum of the flavonoids (component 4), concentration of guttiferone E (component 5), besides of the eight antibacterial concentrations of the red propolis extract (component 6) 500 µg/mL, (component 7) 375 µg/mL, (component 8) 280 µg/mL, (component 9) 210 µg/mL, (component 10) 158 µg/mL, (component 11) 118 µg/mL, (component 12) 88 µg/mL, and (component 13) 66 µg/mL. The result of bacterial-growth inhibition of the antibacterial test received the notation 1 and bacterial-growth results of the received the 0 notation. The PCA-X plot and PCA loading plot were analysed separately for the phenol compounds tested against all antibacterial concentrations of the red propolis extract. The Hotelling T^2^ paired statistical test, Anova test (F) and p value (0.05) were used to compare the relationship between phenolic variations and bacterial growing in specific months tested. In the Hotelling T^2^ paired test, propolis B assumed control group in relation to propolis A and propolis C, because there was no bacterial growing at the tested-concentration range.

Other correlation analysis were stablished between some specific flavonoids (Guttiferone E, Guttiferone B, formononetin, isoliquiritigenin, naringenin, Biochanin A, pinocembrin, daidzein, genistein, galangin, luteolin, liquiritigenin, pinobanksin) quantified by chromatographic techniques during the months of the seasonal study and the anti-staphylococcal activity. A new matrix 36 × 14 (36 samples of the seasonality of propolis A, propolis B, propolis C and 13 important phenol compounds quantified by chromatographic techniques and the last variable was the anti-staphylococcal activity, which was transformed in weight function). The phenolic compound concentrations were normalized and the eight antibacterial concentrations were transformed in weight function (wf) as follows: 0.25 to the concentration 500 µg/mL, 0.30 to the concentration 375 µg/mL, 0.45 to the concentration 280 µg/mL, 0.60 to the concentration 210 µg/mL, 0.75 to the concentration 158 µg/mL, 0.80 to the concentration 118 µg/mL, 0.90 to the concentration 88 µg/mL and 0.95 to the concentration 66 µg/mL. Each sample of the months of seasonal study received values of the weight function between 0 and 5, according to the sum of the concentrations that inhibited staphylococcal growth. Concentrations that did not inhibit received 0 value. A classification of the seasonality samples was performed based on the INMET data for the months of intense rains and months of intense sun and the OPLS-DA, PLS-DA and VIP PLS-DA graphs were obtained to detect the phenolic compounds are influencing the activity anti-staphylococcus. The experiment was validated by the ANOVA test, with p value (0.05) and values Q2 > 0.62 and R2 > 0.67.

## Supplementary information

Supplementary Information

 Supplementary Dataset 1 - 11.

## Data Availability

The authors declare the availability of the research data when requested.
