# Supplementary material for: Comprehensive multivariate correlations between climatic effect, metabolite-profile, antioxidant capacity and antibacterial activity of Brazilian red propolis metabolites during seasonal study
Source: Sci Rep. 2019 Dec 4;9:18293. doi: 10.1038/s41598-019-54591-3 (PMC6893030; doi:10.1038/s41598-019-54591-3)
Supplement: Supplementary file 1 — Supplementary Information [file 41598_2019_54591_MOESM1_ESM.pdf]

Comprehensive multivariate correlations between climatic effect, metabolite-profile, antioxidant capacity and antibacterial activity of Brazilian red propolis metabolites during seasonal study

Ticiano Gomes do Nascimento*[1], Rodolfo Elleson Santos Arruda[1], Erika Tayse da Cruz Almeida[1], José Marcos dos Santos Oliveira[1], Irinaldo Diniz Basílio-Júnior[1], Isabel Cristina Celerino de Moraes Porto[1], Adilson Rodrigues Sabino[1], Josealdo Tonholo[1], Alexander Gray[2], RuAngelie Edrada Ebel[2], Carol Clements[2], Tong Zhang[2], David George Watson*[2]

[1] Laboratory of Pharmaceutical and Food Analysis, Postgraduate Program in Pharmaceutical Sciences, Institute of Pharmaceutical Sciences and Postgraduate program of Nutrition, College of Nutrition, Federal University of Alagoas, Campus A. C. Simões, University City, Maceió/Alagoas/Brazil. Postcode: 57072-970. Phone/FAX: 0055 21 82 3214 1155. E-mail: ticianogn@yahoo.com.br*
[2] University of Strathclyde, Department of Pharmaceutical Sciences, Strathclyde Institute of Pharmacy and Biomedical Science, 27 Taylor Street, Glasgow, G4 0NR, UK. Tel.: +44 1415482707; Fax: +44 1415526399. E-mail: d.g.watson@strath.ac.uk* (D.G. Watson)

S1. Experimental

S.1.1 Chemicals and biologicals

The analytical standards caffeic acid, ferulic acid and p-coumaric acid, biochanin A, chrysin, catechin, daidzein, dalbergin, formononetin, genistein, galangin, isoliquiritigenin, narigenin, pinobanksin, Pinocembrin, quercetin, luteolin were purchased form sigma-aldrich and liquiritigenin and epicatechin were purchased from extrasynthese (Lyon Nord, France). The laboratorial standards guttiferone E (isomeric mixture) and guttiferone B (isomeric mixture) were isolated and purified using flash and semi-preparative techniques and identified by NMR 1H and 13C and LC-Orbitrap-FTMS at the Pharmaceutical Analysis Laboratory-SIPBS-University of Strathclyde-UK.
The bacteria and protozoan strains were used from the American Type Culture Collection (ATCC) Staphylococcus aureus (ATCC 25293) and Pseudomonas aeruginosa (ATCC 27853) and Trypanosoma brucei brucei S427 a Trypanossoma brucei blood strain form was used. The culture media HMI-9 medium (invitrogen), Mueller Hinton agar, BHI agar, nutrient Agar were purchased from Difco® and were used for the microbiological testing and trypomastigotes activity. Suramin, DMSO and resazurin (Alamar Blue) were acquired from sigma-aldrich.
Analytical grade reagents: ethanol, potassium dihydrogen phosphate and dipotassium hydrogen phosphate, formic acid and HPLC grade methanol were purchased from J.T. Baker (Mallinckrodt, Mexico), HPLC grade acetonitrile was purchased from Fisher Scientific (Leicestershire, UK) and Milli-Q grade water was produced in lab.

S.1.2 LC-UV-DAD and LC-ESI-Orbitrap-FTMS

The LC-UV-DAD system consisted of an LC-20AT pump, a column oven (model CTO-20A), a diode array detector (SPDM20A), an autosampler SIL-20A, and a controller module SCL-20A (all from Shimadzu) coupled to a personal computer running the software Shimadzu Lab solution for data acquisition.

The mobile phase was adjusted to the flow rate of 600µL/min of mobile phase (formic acid in H_2O, 1:19, v/v: methanol; A:B) in SB-C18 column (150 x 4.6 mm; 2.7µm) from poreshell® from agilents using gradient mode starting with 36% B, increase to 38% B (4.0 min.), increase to 41% B (7.0 min), increase to 47% B (12 min), increase to 57% B (20 min.), increase to 69% B (31min.), increase to 78% B (38 min.), increase to 90% B (48min.), and the decrease to 36%B (53 min.) and held at 36% B during 5 min (58min.) and new injection can be performed. The column oven was adjusted to temperature of 40°C and wavelengths of UV detector were adjusted to 265, 280, 320, 375nm. The volume injected into the autosampler was 2µL.

The LC-Orbitrap-FTMS system consisted of an Accela 600 HPLC system combined with an Exactive (Orbitrap) mass spectrometer from Thermo Fisher Scientific (Bremen,Germany) including

on-line DAD(200–600nm) and UV at 280 nm analysis. The MS detection range was from 100 to 1200 m/z set at 30,000 resolutions and the scanning was performed under ESI negative polarity mode with capillary temperature was 250°C. The mobile phase (0.1% of formic acid in H_2O: 0.1% of formic acid in Acetonitrile; A:B) in flow rate of 300µL/min and ACE® C18 columns (100 x 4.6mm; 3µm) from (Hichrom, Reading UK) was used in separation of the phenolic compounds from red propolis. The gradient elution was programmed as follows: 0-6 min linear gradient 30% to 45% of B, 6-14 min linear gradient 45% to 75% of B, 14-20 min linear gradient 75% to 100% of B, 20-51 min at 100% of B for elution of the guttiferones and cleaning of the column, 51-54 min decreasing in B to 45%, 54-55 linear gradient 30% of B, 55-60min isocratic condition with 30% of B to re-equilibration of the column for next run. The injection volume was 10 µL.

S.1.3 Chromatographic Methods validation

S.1.3.1 LC-UV-DAD

In LC-UV-DAD analysis the analytical standards of flavonoids were weighed (2.00 mg) and solubilized in 10 mL of a solvent system (methanol:H_2O; 7:3 v:v) homogenized and filtered in PTFE syringe filters of 0.22 µm. These stock solutions were diluted for the concentrations of 0.15, 0.25, 0.50, 1.00, 5.00, 7.50, 10.00, 12.50, 15.0 µg/mL for establishment of calibration curve during three different days. The concentrations of 5.00, 10.00 and 15.00 µg/mL were used as Quality Control Samples (QCS´s) during the intra-day and inter day precision and accuracy tests during three different days. The validation assays following ICH guidelines requirements (ICH, 2005)[90]. All stock solutions were stored in the refrigerator at the temperature of 2 ºC.

The suitability of the method was performed by injection of all standards in mixture at the concentration of 10 µg/mL prior to the work solution of crude extract of red propolis and always in triplicate. It was necessary to match the peaks of the identifiable compounds and avoid uncertain in retention time variation in this complex matrix. Robustness and parallelism were performed using aliquots of 250 µL, 500 µL and 750 µL of the analytical standards of quercetin and chrysin (200 µg/mL), which were added to get a concentrations of 5.00, 10.00 and 15.00 µg/mL of the quercetin and chrysin in final solution of the crude extract (250 µg/mL) of red propolis. This procedure was used for determination of inter-day precision and accuracy by standard addition in three different days and during the seasonal study. These tests were important establish the linearity of the dilution outliers of the calibration curve.

Crude extracts (100 mg) of the seasonality samples were weighed and solubilized in (10 mL) ethanol transferred to a volumetric flask and then an aliquot of 250 µL was diluted with methanol (10 mL) in volumetric flask to obtain work solutions (250 µg/mL) of the red propolis crude extract, which were directly injected in LC-UV-DAD. Aliquots of 250 µL, 500µL and 750 µL of the quercetin and chrysin analytical standards (200 µg/mL) were added to the final dilution of red propolis extract to obtain concentrations of 5.00, 10.00 and 15.00 µg/mL of quercetin and chrysin. Final concentration (250 µg/mL) of the crude extracts of red propolis also were directly injected in chromatographer during the seasonal study using LC-UV-DAD. These preparations were performed in all validation and seasonal samples to evaluate the suitability of the method during validation study and to serve as reference peaks during running of the seasonal samples. All seasonal samples were prepared freshly prior to be injected in LC-UV-DAD.

S.1.3.2 LC-Orbitrap-FTMS

In LC-Orbitrap-FTMS analysis, analytical standards of flavonoids or guttiferones (10.0 mg) were exactly weighed and solubilized in volumetric flask of 5 mL with methanol (stock solutions at 2000.0 µg/mL) and then the work solutions were prepared using a methanol. Aliquots of each standard were diluted for same volumetric flask in a pool of phenolic compounds (a mixture of standards) to get concentrations of 10,000.0; 5,000.0; 2,000.0; 1,000.0; 500.0; 100.0; 50.0; 10.0 and 1.0 ng/mL and injected directly into the LC-Orbitrap-FTMS. Calibration curve, Accuracy, intra and inter-day precision were performed following ICH guidelines and USP requirements during a six different days and 1 ng/mL was determinate the limit of quantification.

The crude extracts (100 mg) of 36 samples of seasonal study were solubilized in ethanol (5 mL) to obtain stock solution of 20 mg/mL. Two work solutions were prepared at the concentration of 200 µg/mL (for flavonoids in low concentration) and 10 µg/mL (for phenolic compounds in high concentration) which were injected directly into the LC-Orbitrap-FTMS. The samples have been stable for a period of this study. The stock solutions of the standards and the samples of seasonality were stored in freezer (-20 ºC) and refrigerator (8 ºC), respectively, during a period of 30 days without degradation or decreasing in the area integration. All workday calibration curve was performed at the linear range of concentration and two replicates. The validation assay follows also follows the ICH guidelines[90] and FDA[91] requirements.

S.1.4 Determination of total phenolic content and total flavonoids content

The determination of phenolic acids was standardized according the Folin-Ciocalteu method as follows: Gallic acid (100 mg) was weighed and transferred to volumetric flask and then solubilized in (10mL) distilled water to obtain a “stock solution” of 10,000 µg/mL. An (1mL) aliquot was removed of the stock solution and solubilized in water to a flask of 10 mL to obtain “work solution” of 1000 µg/mL. Aliquots of 20, 25, 40, 50, 60, 70, 75, 80 and 100 µL were removed from work solution and transferred to (10mL) volumetric flasks and then was added 250 µL of Folin-Ciocalteu, 6 mL of distilled water and stirred for 1 minute, then an aliquot of 750 µL sodium carbonate (0.3 mg/mL) was added and stirred for 30 seconds. Distilled water was added to the volumetric flask to obtain concentrations 2.0, 2.5 4.0 5.0, 6.0, 7.0, 7.5, 8.0 and 10 µg/mL. The samples were stored in the place protected from light. Direct readings in UV were taken every 30 minutes over a period of 2 hours (30, 60, 90 and 120 minutes) the UV-VIS equipment at a wavelength of 750 nm.

The stock solutions (10 mg/mL) and (1 mg/mL) of the crude extract of propolis were prepared in the same conditions, but absolute ethanol was used to complete solubilisation. Aliquots of 250, 350, 500, 650 and 750 µL of the working solutions (1 mg/mL) were taken and transferred to the (10 mL) volumetric flasks and then was added 250 µL of Folin-Ciocalteu, 6 mL of distilled water and stirred for 1 minute, and then an aliquot of 750 µL of sodium carbonate (0.3 mg/mL) was added and stirred for 30 seconds. Distilled water was added to the volumetric flask to obtain concentrations 25, 35, 50, 65 and 75 µg/mL (working solutions). The samples were stored protected from light. The UV-Vis absorbance measurement were taken every 30 minutes over a period of 2 hours (30, 60, 90 and 120 minutes) the UV-VIS equipment was set at a wavelength of 750 nm. The assays were performed in triplicate and the total phenolic content was determinate. These methods were previously validated in the laboratory. Quality control samples of 35, 50 and 65 µg/mL were used for the precision and accuracy tests.

An alternative method also was standardized in laboratory using direct absorbance measurement using UV-Vis spectrophotometer set at the 280 nm wavelength. Determination was based on the concentration of catechin analytical standard using in an external calibration. Catechin analytical standard (20 mg) was weighed and transferred to a volumetric flask and solubilized in absolute ethanol (10 mL) to obtain a stock solution (2000 µg/mL), which was diluted to the concentrations of 1, 2, 4, 5, 6, 8, 10, 12, 15 and 20 µg/mL. Then, submitted to UV-Vis absorbance measurement at 280 nm. The red propolis extracts were previously submitted to desiccation in infrared oven at 105 °C during 15 minutes. Crude extracts (100 mg) of red propolis were exactly weighed and solubilized with ethanol 96 °GL in a volumetric flask (10 mL) to obtain a concentration of 10 mg/mL (Stock solution). The direct measurement were taken by UV spectrophotometry at the maximum wavelength (280 nm) after previous dilution of the sample to a concentration of 5, 10, 20, 30, 40, 50, 60 and 70 µg/mL (working solutions). The assays were performed in triplicate and the total flavonoid was determinate. These methods were previously validated in the laboratory. Quality control samples of 40, 50 and 60 µg/mL were used for the precision and accuracy tests (**Supplementary dataset 1**).

S.1.5 Determination of Final Concentration in Red Propolis Extracts

A) LC-DAD-UV and LC-Orbitrap-FTMS

The values of area obtained from the chromatograms integrations in the samples of propolis red extracts (Propolis A, B and C of the 12 seasonal months) were substituted in the variable (Y), of equation 1 (Y = AX + B), to find the value of concentration (X) correspond to each flavonoids or guttiferones quantified in diluted samples of these experiments. The percentile concentration (%C) of flavonoids or guttiferones quantified in red propolis extracts was performed using equation 2 and expressed as percentage and follows:

$$Y = A \times X + B \ (1)$$

$$\%C = \frac{X}{M} \times 100 \ (2)$$

Where: (Y), corresponds to the absorbance values. (B), corresponds to the linear coefficient of the line. (A), corresponds to the angular coefficient of the line. (X), corresponds to the concentration (µg/mL) of the diluted samples (working solutions). (M), corresponds to the concentration (µg/mL) of the red propolis extract introduced directly into the liquid chromatograph, being 250 µg/mL for the LC-DAD-UV and 200 µg/mL or 10 µg/mL for the LC-Orbitrap-FTMS assay. It was possible to calculate the final concentration of flavonoids or guttiferones in the initially weighed sample (100 mg) of the red propolis extract from the percentage concentration (%C) (**Supplementary dataset 1**) and then, the result were expressed graphically.

B) Determination total phenolic content and total flavonoids content

The values of the absorbance (Y) obtained by UV-VIS spectrophotometry with the propolis samples (Propolis A, B and C from the 12 months studied) were substituted in the variable (Y), from equation 1 (Y = AX + B) for find the concentration value (X) corresponding to Gallic acid or Catechin quantified in diluted samples of these

experiments. The percent concentration (%C) of Gallic acid or catechin quantified in the extracts of red propolis was performed using equation 2 and expressed as percentage and follows:

$$Y = A \times X + B \ (1)$$

$$\%C = \frac{X}{M} \times 100 \ (2)$$

Where: (Y) corresponds to the absorbance values. (B), corresponds to the linear coefficient of the line. (A), corresponds to the angular coefficient of the line. (X), corresponds to the concentration (µg/mL) of the diluted samples (working solutions). (M), corresponds to the concentration (µg/mL) of the red propolis extract analysed in the UV-VIS spectrophotometer, being 25, 35, 50, 65 and 75 µg/mL for the total phenolic acid content, and 40, 50 and 60 µg/mL for the total flavonoid assay. These concentrations were used as quality control samples in the assay of determination of total phenolic acid content or total flavonoid content, respectively. It was possible to calculate the final concentration of total phenol content or total flavonoid content in the initially weighed samples (100 mg) of the red propolis extract from the percentage concentration (%C) (**Supplementary dataset 1**) and then, the results were expressed graphically.

S.2 Results

S.2.1 Method validation for LC-UV-DAD and LC-ESI-Orbitrap-FTMS

The flavonoids LC-Orbitrap-FTMS quantification method presented good values of precision and accuracy in the specific concentration range for each phenolic compound (Table S2 online). The intra-day precision data were less than 8.00% variation (Guttiferone E 4.50%, Guttiferone B 6.60%, formononetin 4.60%, isoliquiritigenin 3.40%, biochanin A 3.10%, daidzein 6.80%, genistein 3.60%, naringenin 5.3% galangin 7.8% and Pinocembrin 3.80%). The intermediate accuracy data were lower than 10%, except for pinocembrin (14.40%) and naringenin (13.30%). The accuracy data revealed low values than 10.00%, except to pinocembrin (11.90%). The accuracy data revealed values lower than 10%, except for pinocembrin (11.9%). Data of intermediate accuracy and accuracy did not exceed 10% in the limit of quantification, except guttiferone E 16.20% and 15.70%, guttiferone B 13.50% and 6.70% and formononetin 12.20 and 9.4%, respectively. The method proved to be robust for quantification of flavonoids and guttiferones in samples of crude extracts of red propolis at different times of the year.

The method of determination of specific flavonoids using LC-DAD-UV showed linear in the range of 0.15 to 15.00 µg/mL with intra-day precision < 5.00%, inter-day precision < 8.00% and an accuracy < 13.00% to the analytical standards tested (liquiritigenin, daidzein, pinobanksin, luteolin, genistein, isoliquiritigenin, formononetin, pinocembrin and Biochanin A) and quercetin and chrysin were used as internal standard for this method.

S.2.2 Determination of total phenolic content and total flavonoids content

The methods of determination of total phenolic acids using Folin-Ciocalteu and UV-vis at 280 nm methods were previously validated using three quality control samples. The total phenolic acids method presented intermediate precision of 4.30% and an accuracy of 4.53%. The method was linear in the range of 2.00 to 10.00 µg/mL (y = 0.1059x + 0.066) for the gallic acid analytical standard. The UV-vis reading method to catechin analytical standard presented an intra-day precision of 5.50%, intermediate precision of 5.98% and an accuracy of 7.06%. The assay showed an intermediary precision of 2.64% and accuracy of 3.54% with a linearity range between 1.35 to 20.00 µg/mL (y = 0.0687x + 0.0069; R2 = 0.9998) for the catechin standard. The crude extract presented intermediate precision of 6.90% and an accuracy of 8.80% and a linearity in the range of 5.00 to 70.00 µg / mL (y = 0.01126x + 0.0059; R2 0.9998). The UV-vis method and method of determination of total phenol compounds met the regulatory requirements for validation of analytical and bioanalytical methods. The complexity of the red propolis crude extracts justifies this greater variability (7.06% and 8.80%, and therefore 3.80%) higher than the limits recommended by regulatory agencies 29 in relation to the tests with the analytical standards of gallic acid and catechin.

The propolis A (Ilha do Porto apiary), propolis B (Primavera apiary) and propolis C (Paripueira apiary) had an average concentration of 26 mg, 30 mg and 33 mg of catechin/100 mg of Red Propolis Extract, respectively. The seasonal variation of Propolis A presented higher concentrations of flavonoids for the July, August and September months (34.5 mg of catechin/100 mg of Red Propolis Extract), but the March, May and December months presented low average concentration in the year (20 mg of catechin/100 mg of Red Propolis Extract). The Propolis B showed higher concentrations in the months of December, January and February, the concentrations reached about 33 mg of catechin/100 mg of Red Propolis extract, although the months of June and July obtained the lowest results (23 mg of catechin/100 mg of Red Propolis extract). The Propolis C showed lower results in the flavonoid concentrations (average concentration of 23 mg of catechin/100 mg of extract) can

be observed for the months of April, May and June, which approached 13 mg/100 mg of Red propolis of Extract (Figure S2 online; Graphs A.1, B.1 and C.1).

Propolis A, B and C showed an interesting total phenols profile with an average concentration of 17.85, 23.53 and 18.23 mg of gallic acid/100mg of propolis extract during a year cycle. The higher concentration was observed during August, November and July, (23.58 mg, 27.64 mg and 24.1 mg of gallic acid/100 mg propolis extract, respectively to Propolis A, B and C), respectively; and a decrease in concentration were observed in October (propolis A), June (propolis B) and April (propolis C) (14.87, 17.62 and 12.90 mg of gallic acid/100 mg of propolis extract, respectively) (Figure S2 online; Graphs A.2, B.2 and C.2).

Supplementary reference

90. ICH, Q2 (R1). "Validation of analytical procedures: text and methodology", ICH harmonised tripartite guideline. International conference on harmonisation of technical requirements for registration of pharmaceuticals for human use, Chicago, USA, https://www.ich.org/fileadmin/Public_Web_Site/ICH_Products/Guidelines/Quality/Q2_R1/Step4/Q2_R1__Guideline.pdf (2005).
91. FDA, Analytical Proceduresand Methods Validation for Drugs and Biologics Guidance for Industry. In: Search for FDA Guidance Documents. Food and Drug Administration. 2015. https://www.fda.gov/media/87801/download Accessed 06 Oct 2019.

Author contributions statement

T.G.N. conceived the experiment(s), T.G.N, R.E.S.A., J.M.S.O., E.T.C.A., A.R.S., T.Z., C.J.C and D.G.W. conducted the experiment(s), T.G.N, R.E.S.A., J.M.S.O., E.T.C.A., I.C.C.M.P, A.I.G., R.E.E, I.D.B.J., J.T., A.R.S., T.Z. and D.G.W. analysed the results. T.G.N., J.M.S.O., R.E.E and D.G.W. gathered the literature data. T.G.N, T. Z. and A.R.S prepared the PLS-DA and PCA figures. All authors reviewed the manuscript.

Competing interests

The authors declare that there are no competing interest including financial or non-financial.

Availability data

The authors declare the availability of the research data when requested.

SUPPLEMENTARY TABLES

Table S1. Identification and confirmation of some markers of the Brazilian red propolis extracts using LC-ESI- Orbitrap-FTMS

Peak	RT (min.)	$[M-H]^-$ (m/z)	MW	Formulae	Compound
1	2.95	179.05	180.16	$C_9H_8O_4$	Caffeic acid
2	2.98	193.05	194.18	$C_{10}H_{10}O_4$	Ferulic acid
3	3.00	178.05	179.05	$C_9H_8O_4$	Umbelic acid
4	3.04	163.02	164.16	$C_9H_8O_3$	p-coumaric acid
5	3.10	475.12	476.43	$C_{23}H_{24}O_{11}$	7-O-beta-glucopyranosyl-4'-hydroxy-5-methoxyisoflavone
6	4.50	461.10	462.40	$C_{22}H_{22}O_{11}$	6-Methoxyluteolin 7-rhamnoside
7	7.05	269.08	270.24	$C_{15}H_{10}O_5$	Genistein
8	7.35	285.03	286.24	$C_{15}H_{10}O_5$	Kaempferol
9	8.04	289.07	290.27	$C_{15}H_{14}O_6$	Cathechin
10	8.28	287.05	288.25	$C_{15}H_{12}O_6$	Dalbergioidin
11	8.83	289.07	290.27	$C_{15}H_{14}O_6$	Epicatechin
12	8.95	253.04	254.24	$C_{15}H_{10}O_4$	Daidzein
13	9.70	255.06	256.27	$C_{15}H_{12}O_4$	Liquiritigenin
14	10.5	283.03	284.26	$C_{16}H_{12}O_5$	2´-Hydroxyformononetin
15	11.3	331.08	332.30	$C_{17}H_{16}O_7$	Evernic acid
16-17	11.9	271.06	272.25	$C_{15}H_{12}O_5$	Narigenin / Pinobanksin
18	12.4	285.07	286.24	$C_{15}H_{10}O_6$	Calycosin
19	12.5	301.07	302.24	$C_{15}H_{10}O_7$	Quercetin
20	13.1	522.15	523.56	$C_{32}H_{26}O_7^+$	Retusapurpurin B
21	13.5	521.15	522.55	$C_{32}H_{26}O_7$	Retusapurpurin A
22	13.6	255.06	256.27	$C_{15}H_{12}O_4$	Isoliquiritigenin
23	13.77	267.06	268.28	$C_{16}H_{12}O_4$	Formononetin / Isoformononetin
24	14.2	269.08	270.28	$C_{16}H_{14}O_4$	4,4'-dihydroxy-2-methoxychalcone
25	14.2	269.08	270.32	$C_{16}H_{14}O_4$	(7S)-dalbergiphenol

26	14.66	271.06	272.29	$C_{16}H_{16}O_4$	Vestitol
27	15.10	269.08	270.28	$C_{16}H_{14}O_4$	Pinostrobin
28	15.10	269.08	270.27	$C_{16}H_{14}O_4$	Medicarpin
29	16.2	271.06	272.29	$C_{16}H_{16}O_4$	2',6'-dihydroxy-4'-methoxydihydrochalcone
30	16.2	283.07	284.26	$C_{16}H_{12}O_5$	Thevetiaflavone
31	16.42	283.06	284.26	$C_{16}H_{12}O_5$	Biochanin A
32	16.73	253.09	254.25	$C_{15}H_{10}O_4$	Chrysin
33	16.87	255.10	256.27	$C_{15}H_{12}O_4$	Pinocembrin
34	17.0	539.17	540.56	$C_{32}H_{28}O_8$	3',4'-di-O-benzyl-7-O-(2-hydroxyethyl)-3-O-methylquercetin
35	18.2	285.11	286.32	$C_{17}H_{18}O_4$	(3S)-7-O-methylvestitol
36	18.2	285.11	286.32	$C_{17}H_{18}O_4$	7,3'-Dihydroxy-4'-methoxy-8-methylflavane
37	21.4	425.16	426.71	$C_{30}H_{50}O$	Cycloartenol / α-amyrin / β-amyrin
38	23.6	533.29	534.69	$C_{33}H_{42}O_6$	Hyperibone H
39	25.5	617.35	618.82	$C_{38}H_{50}O_7$	16-hidroxiguttiferone K
40	27.3	511.14	512.50	$C_{30}H_{24}O_8$	Rhuschalcone V
41	32.80	601.35	602.80	$C_{38}H_{50}O_6$	Guttiferone F
42	32.88	601.35	602.80	$C_{38}H_{50}O_6$	Xanthochymol
43	32.90	601.35	602.80	$C_{38}H_{50}O_6$	Guttiferone E
44	34.10	347.22	348.52	$C_{22}H_{36}O_3$	Anacardic acid (6-pentadecylsalycilic acid)
45	34.50	509.27	510.59	$C_{27}H_{38}O_3$	Makassaric acid
46	39.24	669.44	670.91	$C_{43}H_{58}O_6$	Guttiferone C
47	39.24	669.44	670.91	$C_{43}H_{58}O_6$	Guttiferone D
48	39.24	669.44	670.91	$C_{43}H_{58}O_6$	Guttiferone B
49	48.4	401.31	402.60	$C_{26}H_{42}O_3$	19-nor-10-keto-25-hydroxyvitamin D3

RT: Retention time (min), MW: Molecular weight.

Table S2 – Linearity, precision and accuracy of the LC-Orbitrap-FTMS method during the determination of phenolic compounds in seasonality from Brazilian red propolis extract.

Phenolic compounds	Concentration (μg/mL)	Linearity; r^2	Precision				Accuracy	
			Intra-day 1	Intra-day 2	Intra-day 3	Intra-day 4	Inter-day	% Bias
	10.000		8.751	9.222	9.880	10.240	9.523	-4.768
Guttiferone E	5.000	* Y=0.00000008462x+0.00000000106	4.359	4.490	4.763	4.770	4.595	-8.093
	2.000	0.9995	1.990	2.035	2.180	2.185	2.098	4.880
	1.00	* (1/Y = 1/X*a + b)	1.030	1.031	1.045	1.083	1.035	3.550
	0.50		0.5	0.502	0.488	0.492	0.495	-1.018
	10.000		10.156	10.532	10.467	10.344	10.375	3.750
Guttiferone B	5.000	* Y=0.0000004794233x+0.0000000010081	4.855	4.659	4.757	4.806	4.769	-4.620
	1.000	0.9999	0.973	1.001	1.056	1.010	1.009	0.998
	0.500	* (1/Y = 1/X*a + b)	0.501	0.497	0.499	0.498	0.499	-0.210
	2.000		1.955	1.948	1.930	1.990	1.956	-2.206
Formononetin	1.000	Y=145,059,003.2x+22,247,112.7	1.081	1.062	1.099	1.029	1.068	6.762
	0.500	0.993	0.534	0.588	0.589	0.481	0.548	9.600
	0.100		0.075	0.096	0.091	0.100	0.091	-9.423
	2.000		1.996	1.962	1.966	1.996	1.980	-0.998
Isoliquiritigenin	1.000	Y=143,133,826.5x+11,389,569.0	1.000	1.052	1.050	1.011	1.028	2.794
	0.500	0.998	0.520	0.553	0.546	0.493	0.528	5.571
	0.100		0.085	0.094	0.083	0.100	0.090	-9.705
	1.000		0.898	0.977	0.979	0.985	0.960	-4.045
Biochanin A	0.500	Y=123,590,224.8x+6,296,035.2	0.498	0.548	0.540	0.506	0.523	4.587
	0.100		0.114	0.101	0.120	0.097	0.108	8.248
	0.050	0.997	0.047	0.045	0.043	0.045	0.045	-10.463
	1.000		0.990	0.988	0.988	0.987	0.948	-5.231
Naringenin	0.500	Y=81,960,060.0x+2,294,758.1	0.522	0.524	0.523	0.448	0.504	0.809
	0.100		0.088	0.111	0.120	0.100	0.105	4.761
	0.050	0.998	0.045	0.043	0.043	0.049	0.045	-9.674
	1.000		0.937	0.945	0.910	1.018	0.952	-4.756
Daidzein	0.500	Y=130,831,588.5x+2,513,395.2	0.499	0.434	0.470	0.461	0.466	-6.839
	0.100		0.102	0.101	0.100	0.111	0.104	3.584
	0.050	0.997	0.046	0.046	0.047	0.046	0.046	-7.164
	1.000		0.779	0.720	0.981	1.006	0.994	-0.617
Genistein	0.500	Y=114,279,988.2x+4.946.753.8	0.441	0.425	0.458	0.486	0.452	-9.526
	0.100		0.099	0.101	0.100	0.108	0.102	2.033
	0.050	0.995	0.050	0.049	0.050	0.050	0.050	0.006
	0.500		0.418	0.498	0.361	0.485	0.440	-11.934
Pinocembrin	0.100	Y=215,058,167.3x+3,997,734.6	0.101	0.114	0.100	0.100	0.104	3.639
	0.050	0.996	0.048	0.049	0.051	0.050	0.049	-1.136
	0.010		0.011	0.011	0.010	0.010	0.010	3.797
	0.500		0.480	0.457	0.497	0.552	0.497	-0.669
Galangin	0.100	Y=132,781,551.2x+1,499,268.2	0.099	0.101	0.100	0.102	0.1004	0.425
	0.050	0.998	0.050	0.047	0.050	0.050	0.049	-1.602
	0.010		0.010	0.011	0.010	0.010	0.0103	2.569

Table S3. Meteorological data rainfall intensity during the time series between 2009 and 2019.

Month	Cumulative amount of rainfall (mm) per month										
	2009	2010	2011	2012	2013	2014	2015	2016	2017	2018	2019
January	31	188	133	176	53	56	26	160	52	170	197
February	185	138	152	69	17	105	109	62	20	220	75
Marth	162	152	8	135	27	54	58	152	63	86	128
April	260	119	534	72	258	287	18	143	244	485	132
May	581	338	366	87	214	201	225	221	560	145	139
June	374	326	197	262	230	209	471	141	664	35	254
July	191	215	355	228	405	175	190	107	435	84	338
August	279	187	143	20	126	149	181	113	223	62	116
September	88	79	97	29	93	198	25	46	171	28	99
October	9	85	121	80	232	169	54	13	72	1	19
November	19	14	48	14	77	48	2	23	11	53	NA*
December	31	30	16	18	23	33	73	35	57	72	NA*

NA* Unavailable Data at the current data (30/10/2019). Automatic Surface Observation Meteorological Station: Maceio Meteorological Station located 30 km from the Marechal Deodoro city. The data were extracted from the Weather Data Storage Section (SADMET). National Institute of Meteorology (INMET) from the Brazilian Ministry of Agriculture, Livestock and Supply. Brasilia: Accessed in 30 October 2019.

Table S4. Meteorological data solar radiation during the time series between 2009 and 2019.

Month	Average of solar radiation (kJ/m^2) per month										
	2009	2010	2011	2012	2013	2014	2015	2016	2017	2018	2019
January	1006	851	926	267	984	972	937	826	NA*	927	846
February	910	902	936	1155	969	939	922	933	NA*	916	933
Marth	986	924	1000	NA*	952	935	964	844	NA*	896	884
April	831	812	728	NA*	820	787	848	768	NA*	640	759
May	580	719	587	NA*	663	641	715	637	NA*	685	700
June	595	567	655	615	622	644	581	644	NA*	652	592
July	676	674	569	NA*	616	664	590	616	538	620	610
August	695	672	725	592	687	748	701	713	658	793	703
September	885	833	756	867	849	823	855	859	758	864	832
October	1034	886	919	895	922	864	942	914	801	988	952
November	1039	1036	966	1041	912	908	967	969	927	943	NA*
December	993	980	1713	981	974	917	925	950	909	935	NA*

NA* Unavailable Data at the current data (30/10/2019). Automatic Surface Observation Meteorological Station: Maceio Meteorological Station located 30 km from the Marechal Deodoro city. The data were extracted from the Weather Data Storage Section (SADMET). National Institute of Meteorology (INMET) from the Brazilian Ministry of Agriculture, Livestock and Supply. Brasilia: Accessed in 30 October 2019.

Table S5. Ocean Niño Index during the time series between 2009 and 2019.

	**2009**	**2010**	**2011**	**2012**	**2013**	**2014**	**2015**	**2016**	**2017**	**2018**	**2019**
January	-0.79	1.61	-1.44	-0.77	-0.43	-0.39	0.61	2.66	-0.33	-0.88	0.76
February	-0.69	1.35	-1.00	-0.57	-0.41	-0.51	0.52	2.34	0.02	-0.68	0.82
Marth	-0.61	0.98	-0.83	-0.52	-0.16	-0.19	0.56	1.70	0.12	-0.71	0.90
April	-0.26	0.48	-0.67	-0.40	-0.15	0.18	0.79	0.99	0.30	-0.41	0.72
May	0.15	-0.13	-0.43	-0.23	-0.31	0.41	1.01	0.39	0.45	-0.11	0.65
June	0.39	-0.61	-0.30	0.10	-0.35	0.31	1.25	0.05	0.41	0.12	0.54
July	0.52	-1.04	-0.39	0.28	-0.36	-0.03	1.49	-0.44	0.28	0.16	0.39
August	0.51	-1.44	-0.70	0.41	-0.44	-0.08	1.88	-0.63	-0.21	0.04	0.00
September	0.60	-1.67	-0.88	0.30	-0.26	0.21	2.12	-0.65	-0.51	0.39	-0.11
Octuber	0.85	-1.73	-1.09	0.22	-0.24	0.40	2.32	-0.78	-0.60	0.86	*
November	1.40	-1.69	-1.23	0.11	-0.19	0.71	2.67	-0.80	-1.01	0.86	*
December	1.68	-1.65	-1.11	-0.30	-0.22	0.66	2.60	-0.55	-1.04	0.84	*

Source: The data were extracted from the NOAA with permission. The data are in public domain. https://origin.cpc.ncep.noaa.gov/products/analysis_monitoring/ensostuff/detrend.nino34.ascii.txt. Accessed in 30 October 2019.

Table S6. Pearson´s correlation between four analytical methods of phenolic compounds determination used during seasonality study (march/2011-february/2012) of the Brazilian red propolis.

	Phenol Content (PC)			UV-Vis			LC-DAD			LC-MS		
	A	B	C	A	B	C	A	B	C	A	B	C
PC	-	-	-	**0.78**	**0.62**	**0.69**	0.60	0.54	0.61	0.44	0.85	0.56
UV	**0.78**	**0.62**	**0.69**	-	-	-	0.75	0.01	0.67	0.55	0.58	0.64
LCUV	0.60	0.54	0.61	0.75	0.01	0.67	-	-	-	**0.85**	**0.63**	**0.98**
LCMS	0.44	0.85	0.56	0.55	0.58	0.64	**0.85**	**0.63**	**0.98**	-	-	-

A = Propolis A; B = Propolis B; C = Propolis C.

Table S7. Antibacterial activity of the extract of red propolis against *S. aureus* ATCC 25293 stream.

Propolis A Conc.(μg/mL)	Mar.	Apr.	May	Jun.	Jul.	Aug.	Sep.	Oct.	Nov.	Dec.	Jan.	Feb.
500	-	-	-	-	-	-	-	-	-	-	-	-
375	-	-	-	-	-	-	-	-	-	-	-	-
280	-	-	-	-	-	-	-	-	-	-	-	-
210	-	-	-	-	-	-	-	-	-	-	-	-
158	-	-	-	-	-	-	-	-	-	-	-	-
118	-	-	-	-	-	-	-	-	-	-	-	-
88	-	-	-	-	+	-	-	-	-	-	-	-
66	-	-	+	-	+	-	-	-	-	-	-	-
Propolis B Conc.(μg/mL)	Mar.	Apr.	May	Jun.	Jul.	Aug.	Sep.	Oct.	Nov.	Dec.	Jan.	Feb.
500	-	-	-	-	-	-	-	-	-	-	-	-
375	-	-	-	-	-	-	-	-	-	-	-	-
280	-	-	-	-	-	-	-	-	-	-	-	-
210	-	-	-	-	-	-	-	-	-	-	-	-
158	-	-	-	-	-	-	-	-	-	-	-	-
118	-	-	-	-	-	-	-	-	-	-	-	-
88	-	-	-	-	-	-	-	-	-	-	-	-
66	-	-	-	-	-	-	-	-	-	-	-	-
Propolis C Conc.(μg/mL)	Mar.	Apr.	May	Jun.	Jul.	Aug.	Sep.	Oct.	Nov.	Dec.	Jan.	Feb.
500	-	-	-	-	-	-	-	-	-	-	-	-
375	-	-	-	-	-	-	-	-	-	-	-	-
280	-	-	-	-	-	-	-	-	-	-	-	-
210	-	+	-	-	-	-	-	-	-	-	-	-
158	-	+	-	+	-	-	-	-	-	-	-	-
118	-	+	-	+	-	-	-	-	-	-	-	-
88	-	+	-	+	-	-	-	-	-	-	-	-
66	+	+	-	+	-	-	-	-	-	-	-	-
Negative control	-	-	-	-	-	-	-	-	-	-	-	-
Positive control	+	+	+	+	+	+	+	+	+	+	+	+

(+): Blue color in microplate – did not inhibited the bacterial growth; (-): Pink color in microplate – inhibited the bacterial growth; Negative control: Muller Hinton broth medium plus ethanol without *S. aureus*; Positive control: Muller Hinton broth medium plus ethanol with *S. aureus*

Table S8 – Antibacterial activity of the extract of red propolis against *P. aeruginosa* stream.

Propolis A Conc.(μg/mL)	Mar.	Apr.	May	Jun.	Jul.	Aug.	Sep.	Oct.	Nov.	Dec.	Jan.	Feb.
500	-	-	-	-	-	-	-	-	-	-	-	-
375	-	-	-	-	-	-	-	-	-	-	-	-
280	-	-	-	-	-	-	-	-	-	-	-	-
210	-	-	-	-	-	-	-	-	-	-	-	-
158	-	-	-	-	-	-	-	-	-	-	-	-
118	-	-	-	-	-	-	-	-	-	-	-	-
88	-	-	-	-	-	-	-	-	-	-	-	-
66	-	-	-	-	+	-	-	-	-	-	-	-
Propolis B Conc.(μg/mL)	Mar.	Apr.	May	Jun.	Jul.	Aug.	Sep.	Oct.	Nov.	Dec.	Jan.	Feb.
500	-	-	-	-	-	-	-	-	-	-	-	-
375	-	-	-	-	-	-	-	-	-	-	-	-
280	-	-	-	-	-	-	-	-	-	-	-	-
210	-	-	-	-	-	-	-	-	-	-	-	-
158	-	-	-	-	-	-	-	-	-	-	-	-
118	-	-	-	-	-	-	-	-	-	-	-	-
88	-	-	-	-	-	-	-	-	-	-	-	-
66	-	-	-	-	-	-	-	-	-	-	-	-
Propolis C Conc.(μg/mL)	Mar.	Apr.	May	Jun.	Jul.	Aug.	Sep.	Oct.	Nov.	Dec.	Jan.	Feb.
500	-	-	-	-	-	-	-	-	-	-	-	-
375	-	-	-	-	-	-	-	-	-	-	-	-
280	-	-	-	-	-	-	-	-	-	-	-	-
210	-	-	-	-	-	-	-	-	-	-	-	-
158	-	+	-	-	-	-	-	-	-	-	-	-
118	-	+	-	+	-	-	-	-	-	-	-	-
88	-	+	-	+	-	-	-	-	-	-	-	-
66	+	+	-	+	-	-	-	-	-	-	-	-
Negative control	-	-	-	-	-	-	-	-	-	-	-	-
Positive control	+	+	+	+	+	+	+	+	+	+	+	+

(+): Blue color in microplate – did not inhibited the bacterial growth; (-): Pink color in microplate – inhibited the bacterial growth; Negative control: Muller Hinton broth medium plus ethanol without *Pseudomonas aeruginosa* ATCC 27853; Positive control: Muller Hinton broth medium plus ethanol with *Pseudomonas aeruginosa* ATCC 27853.

SUPPLEMENTARY FIGURES

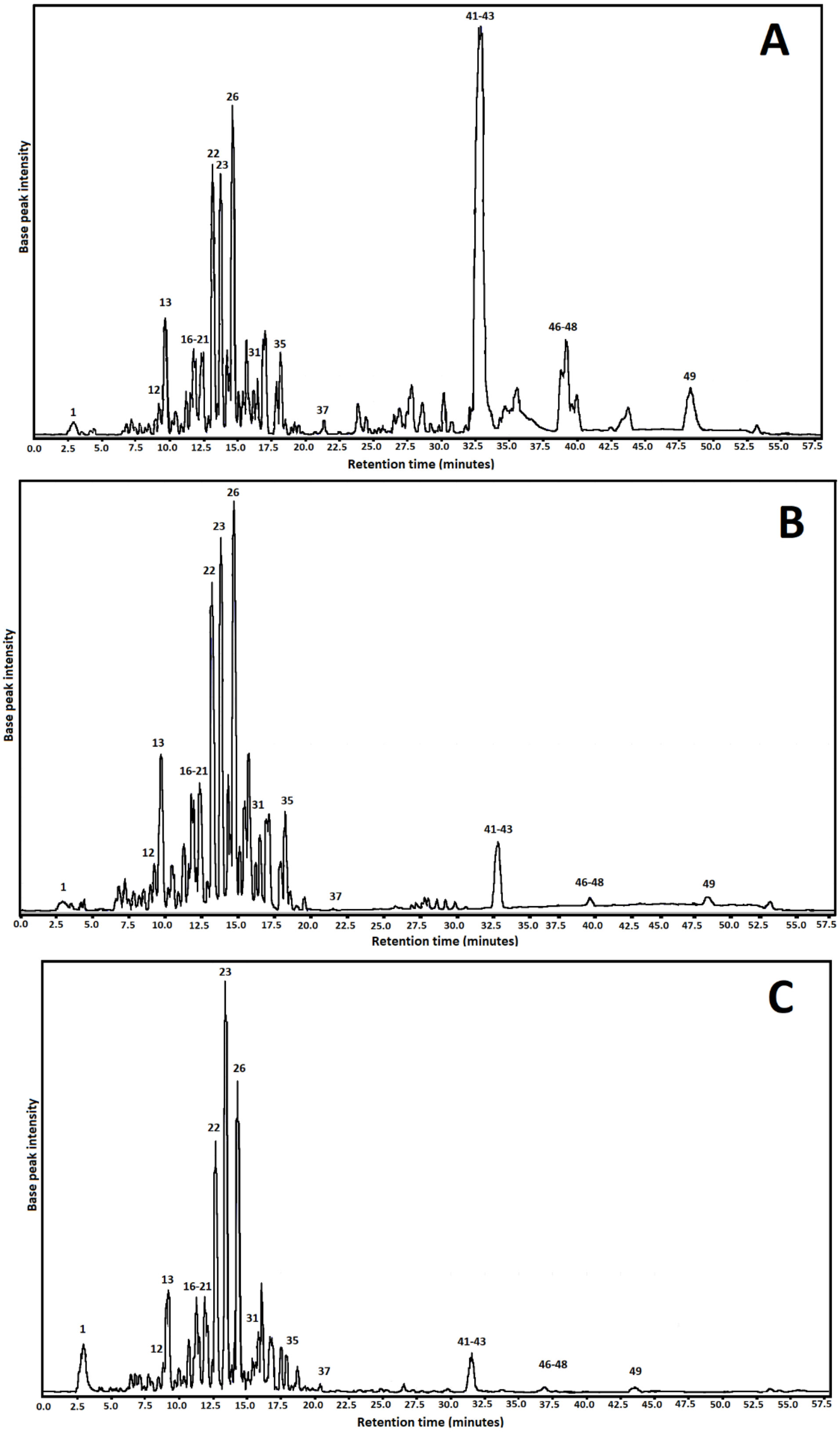

Figure S1. Chromatographic profile of Brazilian red propolis extract using LC-Orbitrap-FTMS. Propolis A from Ilha do Porto apiary (A), Propolis B from Primavera apiary (B) and Propolis C from Paripueira apiary (C).

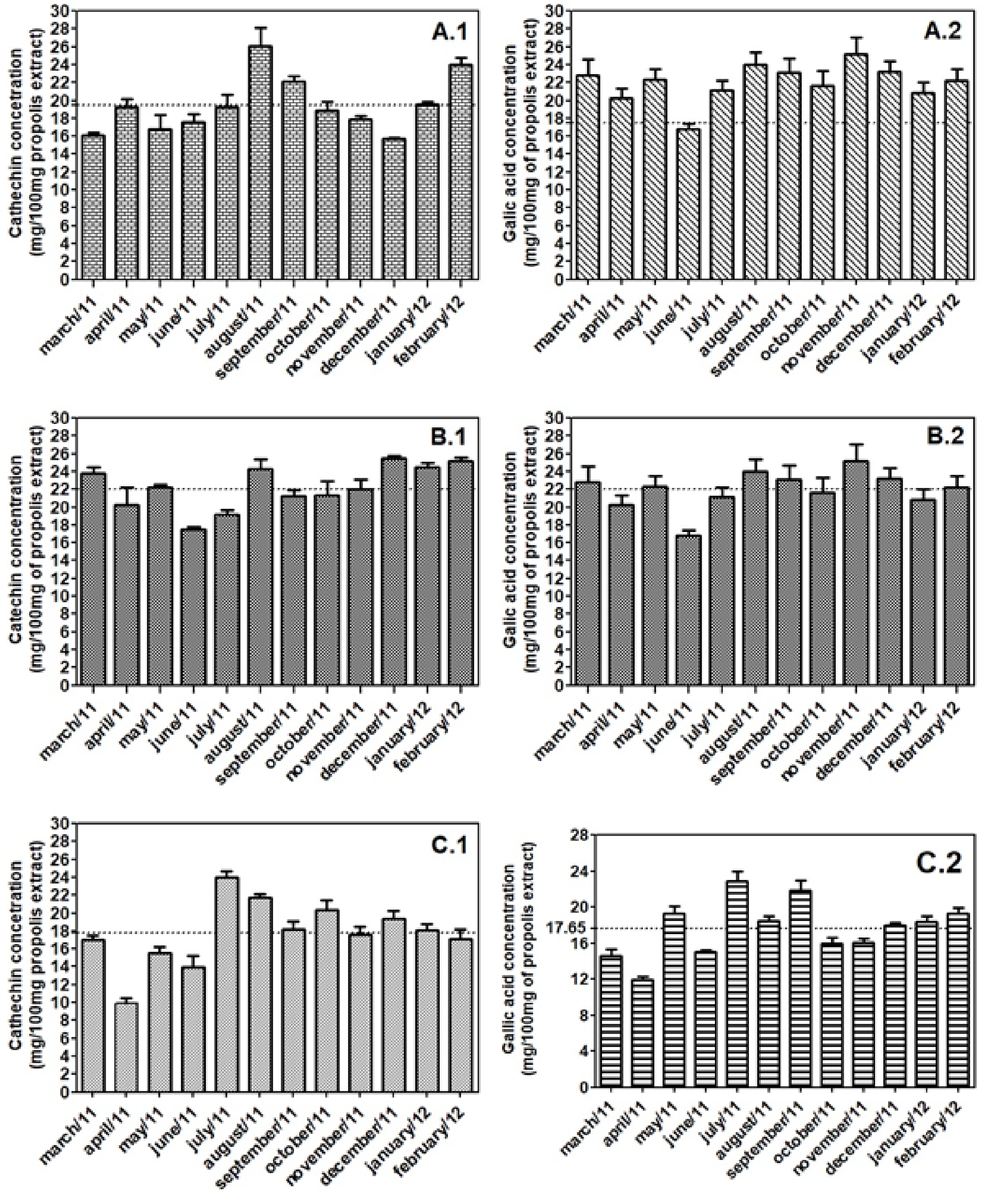

Figure S2. Determination of flavonoids using UV-vis (A.1, B.1 and C.1) and phenolic compounds using Folin-Ciocalteu method (A.2, B.2 and C.2) at the Brazilian red propolis extracts. Propolis A (A.1 and A.2), Propolis B (B.1 and B.2) and Propolis C (C.1 and C.2).

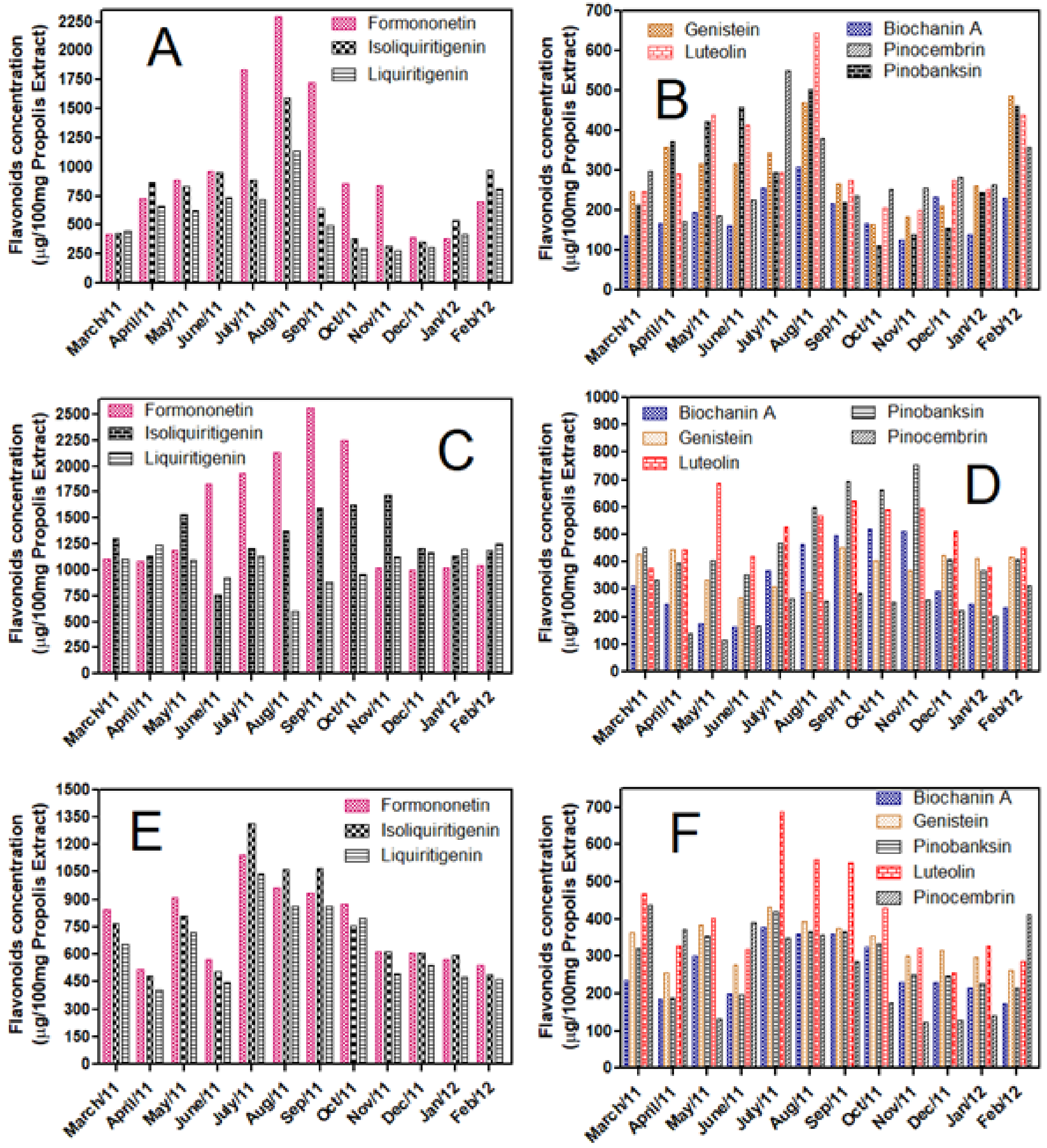

Figure S3. Determination of isoflavonoids and flavonoids of the Brazilian red propolis extracts using LC-DAD-UV. Propolis A (A and B), Propolis B (C and D) and Propolis C (E and F). The concentrations were expressed as amount in microgram of flavonoids present in 100 mg of red propolis extract.

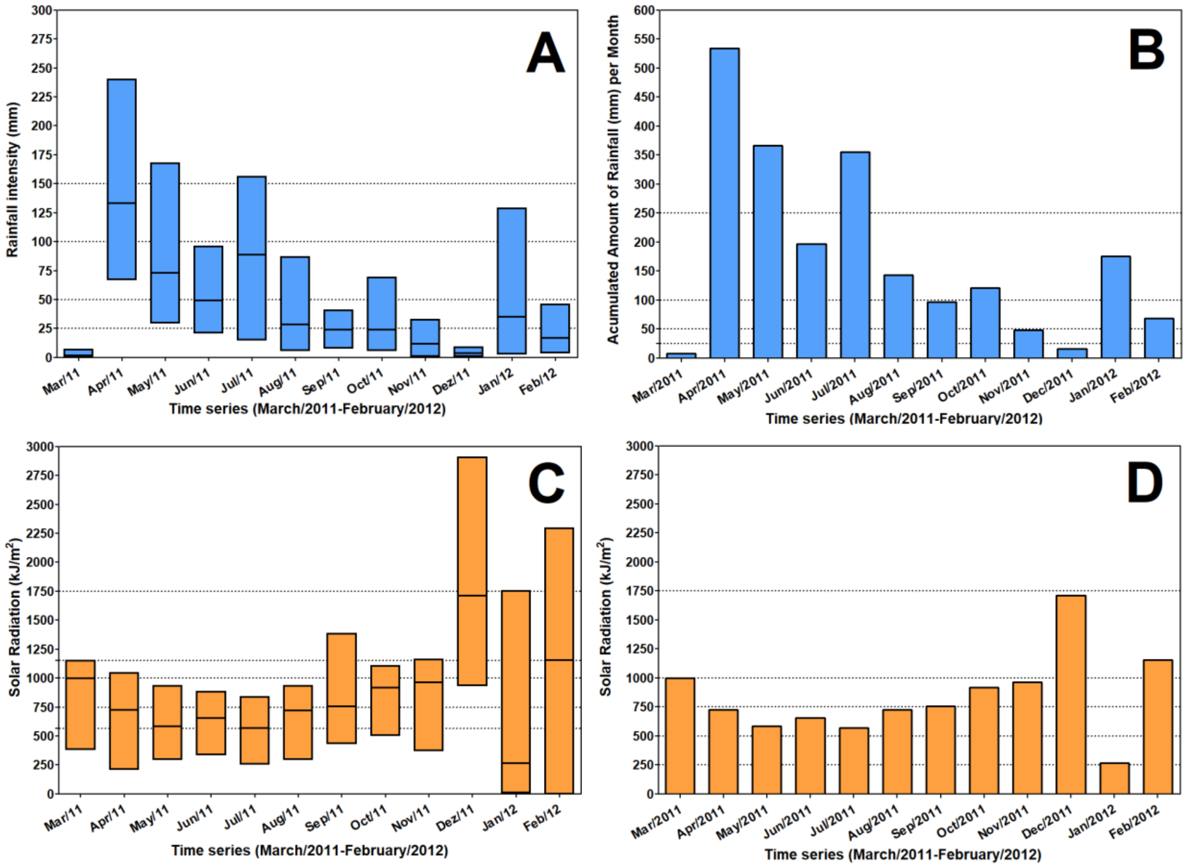

Figure S4. Meteorological data (A) Low-High and mean of rainfall intensity, (B) acumulated amount of rainfall intensity and (C) Low-High and mean of solar radiation and (D) mean of solar radiation during the time series March/2011 and February/2012. Automatic Surface Observation Meteorological Station: Maceio Meteorological Station located 30 km from the Marechal Deodoro city. The data were extracted from the Weather Data Storage Section (SADMET). National Institute of Meteorology (INMET) from the Brazilian Ministry of Agriculture, Livestock and Supply. Brasilia: Accessed in 30 October 2019.

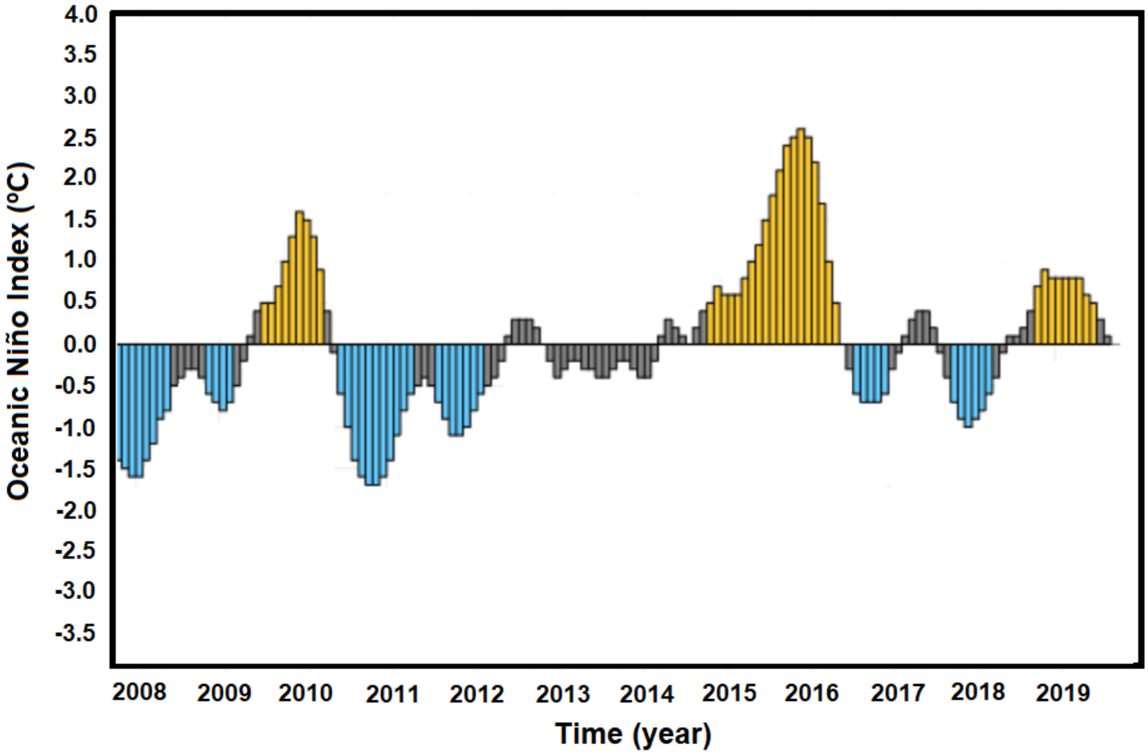

Figure S5. Ocean Niño Index during the time series 2008 and 2019 (up to October) extracted from NOAA agency with permission. The data are in public domain. Accessed in 30 October 2019. The NOAA agency considers El Niño conditions to be present when the Oceanic Niño Index is +0.5°C or higher, indicating the east-central tropical Pacific is significantly warmer than usual. La Niña conditions exist when the Oceanic Niño Index is -0.5°C or lower, indicating the region is cooler than usual.

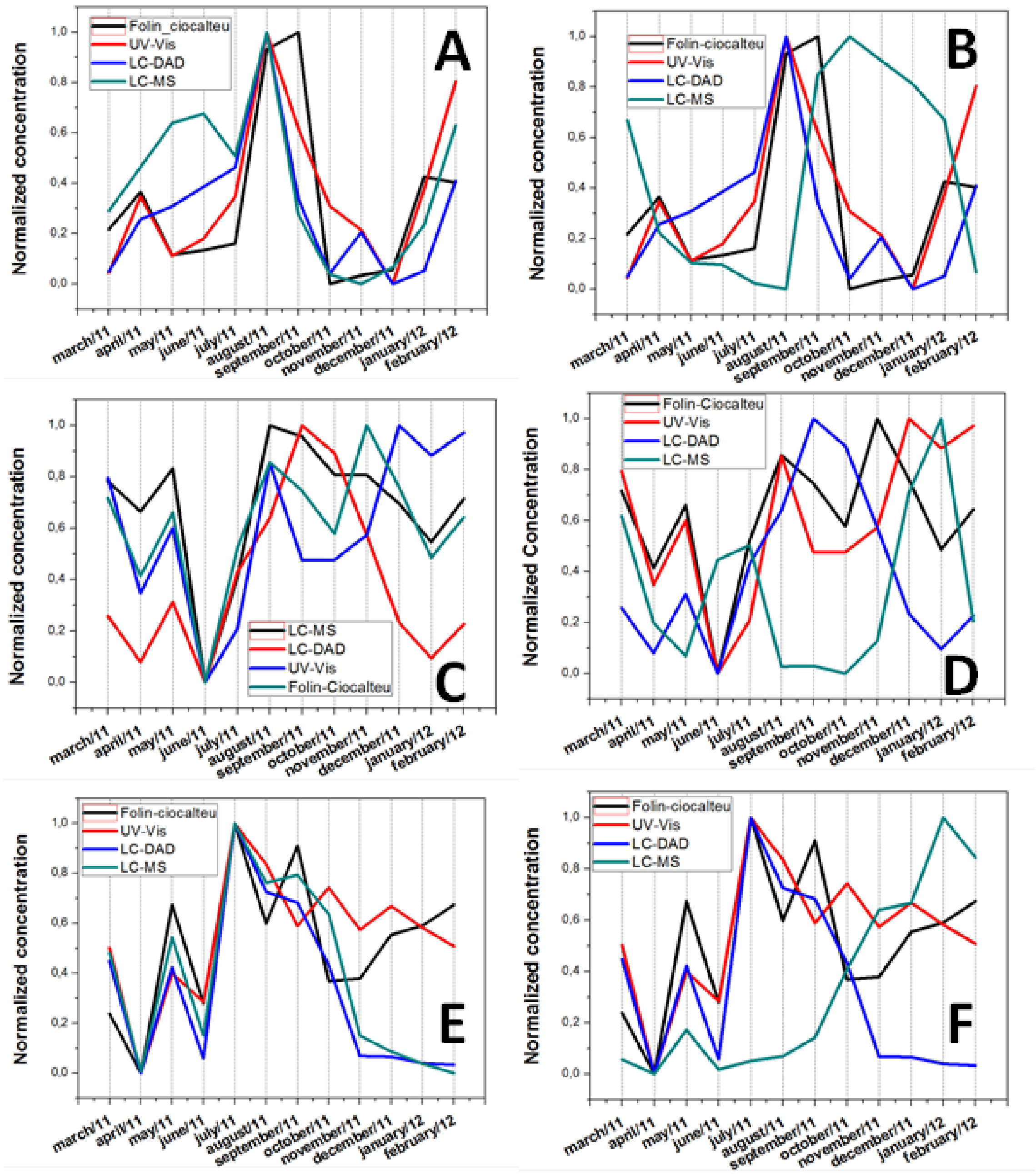

Figure S6. Analytical method correlation between Folin-Ciocalteu method, UV-vis for catechin determination, LC-DAD-UV method and LC-MS method. Propolis A (A and B), Propolis B (C and D) and Propolis C (E and F). Graphs A, C and E were used in Pearson´s correlation among analytical methods. Graphs B, D and F (LC-MS data only) were used in IC_{50} from DPPH method and PCA analysis with MIC tests using *Staphylococcus aureus* and *Pseudomonas aeruginona* strains.

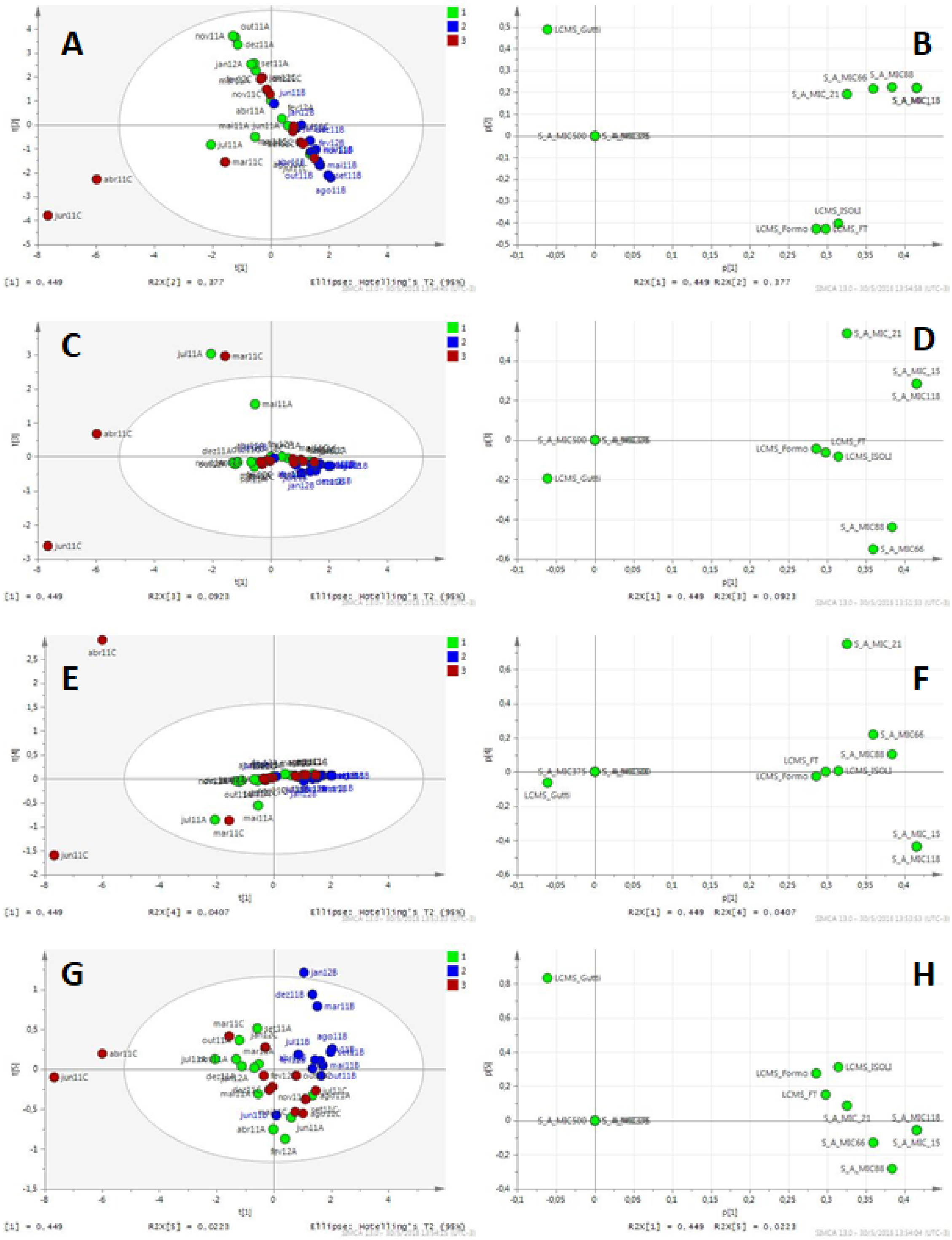

Figure S7. PCA plot and loadings plot showing association between the isoliquiritigenin (A and B), formononetin (C and D), total flavonoids (E and F) and Guttiferone E (G and H) concentrations and *Staphylococcus aureus* in antibacterial test. Propolis A (1), Propolis B (2) and Propolis C (3).

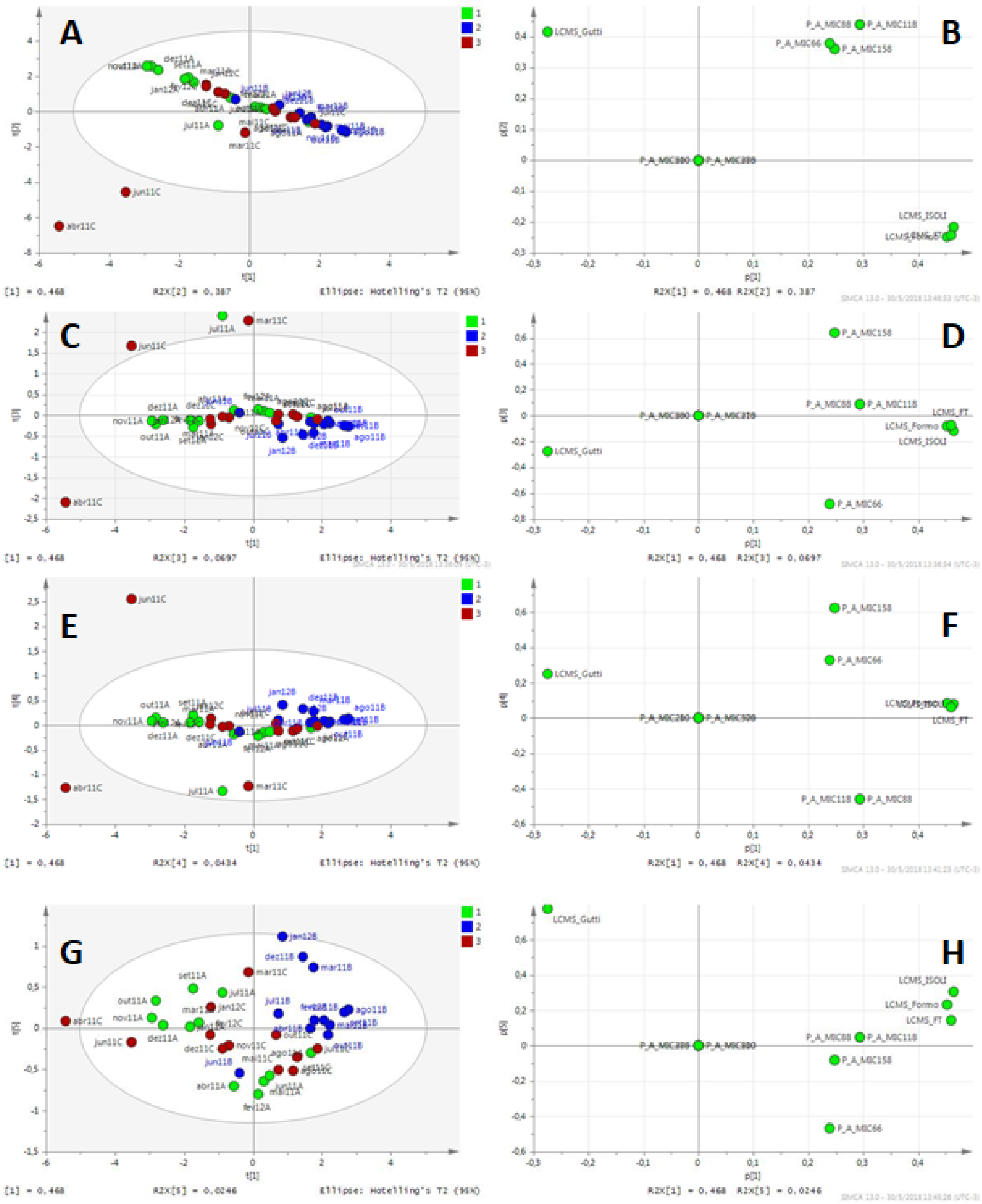

Figure S8. PCA plot and loadings plot showing association between the isoliquiritigenin (A and B), formononetin (C and D), total flavonoids (E and F) and Guttiferone E (G and H) concentrations and *Pseudomonas aeruginosa* in antibacterial test. Propolis A (1), Propolis B (2) and Propolis C (3).

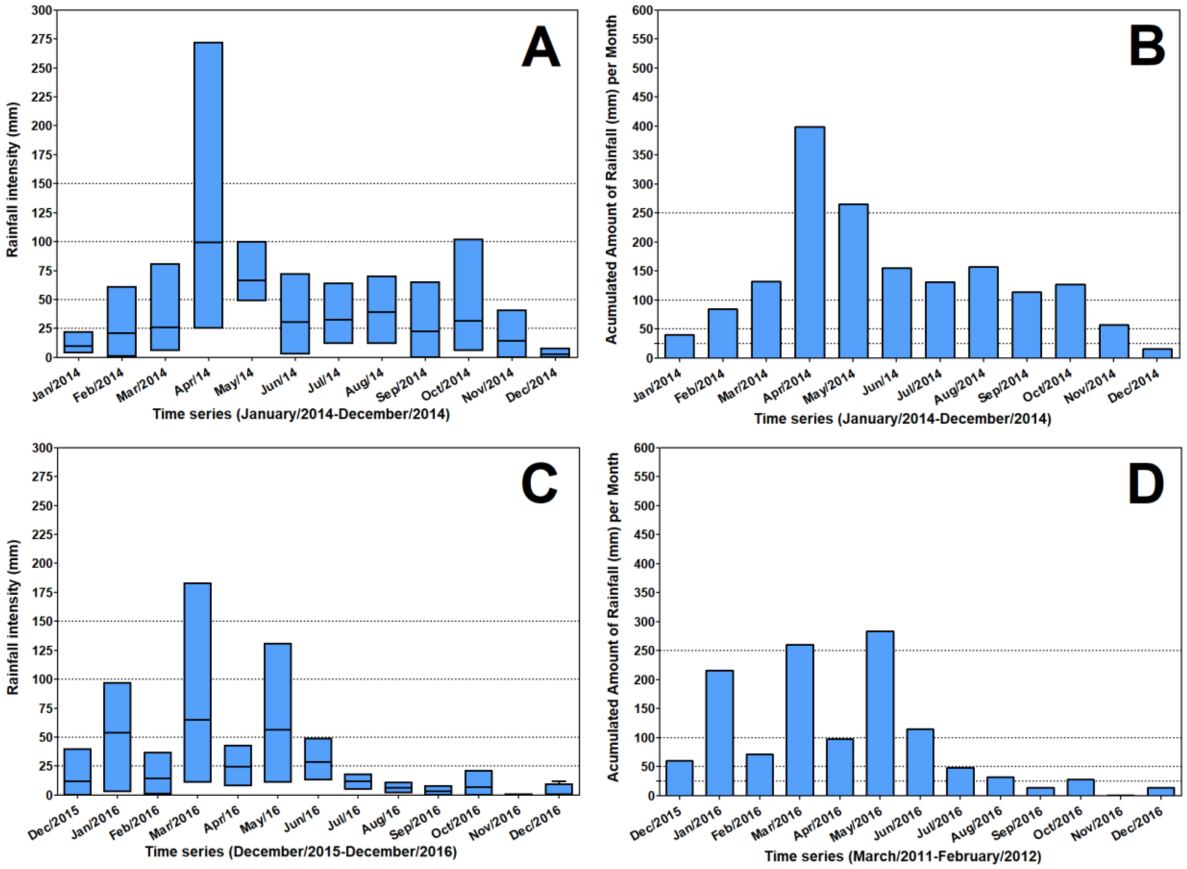

Figure S9. Meteorological data (A) Low-High and mean of rainfall intensity, (B) acumulated amount of rainfall intensity during the time series 2014, (C) Low-High and mean of rainfall intensity and (D) acumulated amount of rainfall intensity during the time series 2015-2016 (December to December). Automatic Surface Observation Meteorological Station: Porto de Pedras Meteorological Station located 50 km from Tamandaré city. The data were extracted from the Weather Data Storage Section (SADMET). National Institute of Meteorology (INMET) from the Brazilian Ministry of Agriculture, Livestock and Supply. Brasilia: Accessed in 30 October 2019.
